# Sorting Out Sorting Nexins Functions in the Nervous System in Health and Disease

**DOI:** 10.1007/s12035-021-02388-9

**Published:** 2021-05-01

**Authors:** Neide Vieira, Teresa Rito, Margarida Correia-Neves, Nuno Sousa

**Affiliations:** 1 Life and Health Sciences Research Institute (ICVS), School of Medicine, University of Minho, Campus Gualtar, 4710-057 Braga, Portugal; 2 ICVS/3B’s - PT Government Associate Laboratory, Braga/Guimarães, Portugal

**Keywords:** Sorting nexins, Nervous system, Evolution, Neurodevelopment, Neurodegeneration, Plasticity

## Abstract

Endocytosis is a fundamental process that controls protein/lipid composition of the plasma membrane, thereby shaping cellular metabolism, sensing, adhesion, signaling, and nutrient uptake. Endocytosis is essential for the cell to adapt to its surrounding environment, and a tight regulation of the endocytic mechanisms is required to maintain cell function and survival. This is particularly significant in the central nervous system (CNS), where composition of neuronal cell surface is crucial for synaptic functioning. In fact, distinct pathologies of the CNS are tightly linked to abnormal endolysosomal function, and several genome wide association analysis (GWAS) and biochemical studies have identified intracellular trafficking regulators as genetic risk factors for such pathologies. The sorting nexins (SNXs) are a family of proteins involved in protein trafficking regulation and signaling. SNXs dysregulation occurs in patients with Alzheimer’s disease (AD), Down’s syndrome (DS), schizophrenia, ataxia and epilepsy, among others, establishing clear roles for this protein family in pathology. Interestingly, restoration of SNXs levels has been shown to trigger synaptic plasticity recovery in a DS mouse model. This review encompasses an historical and evolutionary overview of SNXs protein family, focusing on its organization, phyla conservation, and evolution throughout the development of the nervous system during speciation. We will also survey SNXs molecular interactions and highlight how defects on SNXs underlie distinct pathologies of the CNS. Ultimately, we discuss possible strategies of intervention, surveying how our knowledge about the fundamental processes regulated by SNXs can be applied to the identification of novel therapeutic avenues for SNXs-related disorders.

**Supplementary Information:**

The online version contains supplementary material available at 10.1007/s12035-021-02388-9.

## Introduction

Throughout the intracellular endocytic compartments, the endolysosomal system enables the selective sorting and transport of transmembrane proteins and lipids present at the plasma membrane. These processes involve several adaptor and effector proteins, such as the sorting nexins (SNXs), and can occur through distinct endocytic portals, relying on vesicular and tubovesicular carriers, as well as in maturation, fusion, and fission events. In this manner, endocytosis regulates fundamental processes such as how the cell interacts with surrounding environments controlling nutrient uptake, cell signaling, developmental regulation, cell adhesion, mitosis, antigen presentation, and cell migration. Abnormal function of endocytosis is apparent in distinct disorders, such as cancer, inflammatory conditions, AD, mental retardation and, also of relevance, for pathogen invasion. The central nervous system (CNS) is particularly vulnerable to endolysosomal dysfunction, particularly in aging and in age-associated pathologies, such as neurodegenerative disorders.

The SNXs emerged, in the past decade, as a novel family of proteins that facilitate protein intracellular trafficking and signaling [1, 2], sorting in this manner a wide array of protein cargoes through the endolysosomal system. This family is organized by the presence of a conserved phosphoinositol-binding PX domain [1]. The PX domain binding to phosphatidylinositol phosphate (PIP) enables the association of SNXs with PtdInsP-enriched elements of the endocytic network. In this manner, SNXs redirect protein cargos for retrieval or degradation, playing important roles in protein intracellular trafficking and signaling. SNX1 was the first described mammalian SNX, and it was shown to interact with the epidermal growth factor receptor (EGFR) in a yeast-two hybrid screen [3]. Upon the discovery of the PX domain, bioinformatic approaches and in vitro studies enabled the annotation of other SNXs, whose PX domain displays greater than 50% sequence similarity to SNX1's PX sequence. These SNXs where then assembled as a family [4, 5]. Interestingly, researchers noted the resemblance of SNX1 with a known yeast retromer complex component, Vps5 [6, 7]. The retromer complex, is a “heteropentameric” complex that promotes cargo retrieval from the endolysosomal system back to the plasma membrane, or to the trans-Golgi Network (TGN) [8], conferring a role for SNXs in cargo capture from the degradative pathway. Briefly, the identified metazoan complex consists of a “membrane-associated” sorting dimer (SNX1, SNX2, SNX5, or SNX6), that contains a membrane curvature sensing domain — the BAR domain — and a vacuolar protein sorting trimer (Vps26, Vps29, Vps35). It is important to note, however, that not all SNXs interact with the retromer complex. In fact, to date, 33 mammalian SNXs have been identified [1] from a group of 49 PX-containing proteins encoded in the mammalian genome [2, 9]. The non-SNX PX-domain containing proteins have been subjects of other reviews [2] being majorly associated with cell signal-transduction pathways and protein scaffolding events [9]. Besides the PX domain, some SNXs bear other conserved domains which are involved in a variety of functions, such as membrane curvature-sensing, protein interaction motifs, and signaling motifs. This domain diversity adds “flavors” to this very complex family, where SNXs exert their function by aiding not only in cargo retrieval from degradation, but also in cargo sorting through the recycling and endolysosomal pathways. By doing so, they regulate a panoply of cargoes in distinct types of cells and environmental-contexts. Thus, SNXs can shape how cells adapt to environmental cues, in a manner similar to other protein families that are involved in the maintenance of intracellular trafficking homeostasis [10].

Attention was brought to this family by their emerging association with pathologies of the CNS. Specifically, aberrant expression or autosomal recessive mutations of SNXs have been shown to cause cerebellar ataxia and intellectual disability syndrome [11], as well as to occur in AD [12–15] and in DS [16]. Interestingly, in a DS mouse model, synaptic and cognitive deficits have been rescued by restoring SNX27 hippocampal levels [16]. SNXs dysfunction has also been suggested to be involved in epilepsy [17] and schizophrenia [18–20], and more recently in PD, through their involvement with the retromer complex [21, 22]. Overall, alterations in SNX levels have been associated with endocytic events underlying neuronal function, synaptic plasticity, and shown to impact on complex behaviors like learning and memory, in distinct organisms [1, 2, 11–16, 23, 24]. These recently ascribed roles for SNXs in neuropsychiatric and neurodegenerative disorders justify a reappraisal on how SNXs are sustaining normal brain physiology, and on how this can be perturbed.

Here, we performed a broad integration of the knowledge available concerning SNXs function in the nervous system, both in physiological and pathophysiological settings. We started by spanning the evolution of SNXs as a protein family, focusing on how it is conserved across phyla, surveying species before and after the development of the nervous system, and on how (or if) it correlates with the functional domain complexity of the distinct SNXs family members. We then revisited their involvement in the nervous system development, metabolism and in synaptic plasticity, and focused on how they can trigger pathology, highlighting the described underlying molecular mechanisms. We pinpointed key molecular signature features of SNXs family to shed some light on the role of the endolysosomal system in nervous system dysfunction. Finally, we discuss putative strategies of novel therapeutic interventions.

## The Sorting Nexins

### SNXs Subfamilies: Exploring Functional Implications of Domain Diversity

The hallmark of the SNX family is the presence of a PX domain, a membrane association domain composed of 110 aminoacidic residues, displaying 3 β-strands and 3 α-helices. The PX domain was first identified as a conserved motif in the p40^phox^ and p47^phox^ subunits of the neutrophil NADPH oxidase (phox), a superoxide producing complex [25]. It is involved in the targeting of proteins to cellular membranes by binding to phosphoinositides (PIPs), on the cytoplasmic leaflets of distinct organelles of the endolysosomal system, being thus pivotal for the subcellular localization of PX proteins and thus shaping their function. Distinct studies enabled the assembly of SNX protein as a family, based on the conservation of their PX domain [4, 5]. Off notice, that in addition to SNXs, other proteins contain PX motifs, such as phospholipase D and phosphoinositide-3-kinases [2, 9, 26]. Interestingly, BLAST searches using SNX-PX sequences do not retrieve other non-SNX PX domain containing proteins, supporting that SNX-PX domain evolved independently from other PX domains, and that SNXs might display unique cellular functions. Regarding, PIP binding, distinct studies across phyla highlighted the conserved preference of PX domain for phosphatidylinositol 3-phosphate (PtdIns3P or PI3P) binding, a phosphoinositide enriched in early endosomal limiting membranes, and in this manner, in the sorting of PX domain-dependent protein recruitment to the early endocytic machinery. It is, however, noteworthy that other pools of PI3P can exist in other compartments (such as the plasma membrane) and/or be generated during specific signaling events. In yeast, the PX domain binds almost exclusively to PI3P. In mammals, however, PX domain has been shown to also bind other phosphoinositides [9, 27]. This highlights a broader recruitment of PX-interacting proteins, to a greater diversity of organelles and/or membrane domains, of particular relevance for endosomal trafficking. Still, in vitro reported affinities (Kd values) are quite divergent, namely according to the methods used, ranging from nano- to millimolar values [2]. Some authors have even demonstrated that particular PX domains, such as that of SNX14, do not bind PI3P at all [27]. Furthermore, mounting evidence supports that the PX domain is not only a lipid-binding motif, being also involved in protein-protein interactions, acting as a protein-scaffolding device with ability to shape membrane-association dynamics, and thus subcellular localization, through the dynamic remodeling of its proline loop between helices α-1 and α-2 [2]. The PX domain of some SNXs have can also purportedly shape signaling, by interacting with the cytoplasmic tails of the TGF-β receptor, which was inferred through overexpression studies [2].

**Fig. 1:**
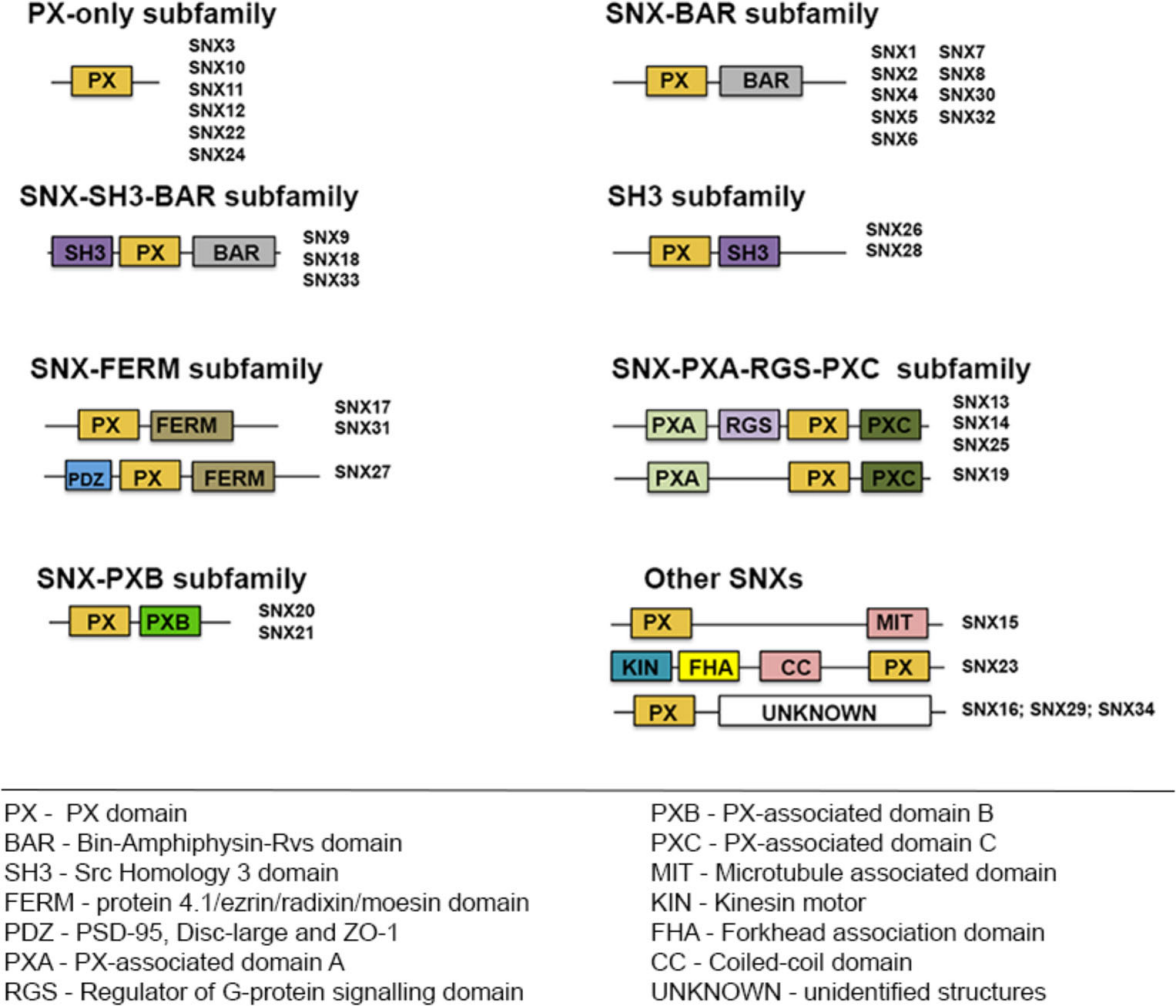
SNXs subfamily classification according to domain organization. Schematic representation of SNXs family organization based on domain conservation or inferred by secondary-structure prediction and sequence comparison. PX domain is present in all annotated members of the family. Other domain abbreviations are represented depicted in the figure. Of notice that within subfamilies some members display unique features, namely in the FERM subfamily the PDZ domain is only present on SNX27; on the PXA-RGS subfamily, a N-terminal transmembrane domain is predicted on SNX13/SNX14/SNX19 but not on SNX25, and RGS domain is absent on SXN19. Representations do not represent a scale

The complexity of the SNX family does not depend solely on its PX-domain PIP and protein binding specificity, but arises also from the existence of a variety of additional conserved domains (Fig. 1), involved in a wide range of cellular processes, spanning from organelle motility, to protein interaction and the downplay of signaling cascades. Phylogenetic analyses have grouped SNXs in distinct PX-containing subfamilies, according to the presence of conserved structural elements and secondary-structures: the PX-only containing proteins; the PX-BAR (Bin/Amphiphysin/RVS) domain containing SNXs; the SNX-FERM (protein 4.1/ezrin/radixin/moesin) proteins, the SNX-PXA-RGS-PXC proteins, and other SNXs [2]. More recent approaches have also highlighted the existence of the PXB domain cluster, the SH3 domain, and the BAR-SH3 clusters within this protein family. SNXs were thus grouped by proposed function taking into consideration the structural domains codified in their sequences (Fig. 1). Still, to date, several SNXs remain to be functionally characterized, or to have their functions validated in vivo, namely in mammals. In an effort to further understand the function of SNXs family members, we describe ahead these distinct clusters.

As stated, PX-only containing SNXs are deprived of additional conserved domains — at least those detected by the available bioinformatic approaches (Fig. 1). Structure wise, PX-only containing SNXs are quite divergent in length and, generally, display sequences with no predicted secondary structures but crucial for their function. This PX-only subfamily is poorly characterized, with SNX3 and SNX12 being the best described. In the fly, worm and mammals, SNX3 (or the SNX3/12 ortholog in lower organisms) has been shown to play pivotal roles in retrograde sorting, through the retromer complex, namely in Wnt signaling [28], and in sorting within the early endosome and the multivesicular bodies (MVBs) [29]. SNX12 has also been demonstrated to play important roles in endosome membrane transport and cargo sorting [30, 31]. Both SNX3 and SNX12 are involved in intraluminal vesicle formation (ILVs) [29, 32], bridging cargo degradation and cargo recycling events. SNX3 and SNX12 role in CNS function and homeostasis regulation will be discussed in further detail in section 3. SNX10, induces giant vacuoles upon overexpression [33], possibly being involved in the fusion of Golgi-derived vesicles and endosomes. This process can be inhibited by SNX11, another PX-only subfamily member [34]. Other SNXs, such as SNX22 and SNX24, whose function remains almost completely unknown, have also been grouped within this subfamily.

The SNX-BAR subfamily is very numerous and displays a C-terminal BAR domain containing group (Fig. 1). These SNXs relevance is supported by distinct studies that explored and dissected their roles in membrane curvature sensing and in retrograde sorting, being the best characterized of all PX subfamilies. The BAR domain — bin-amphiphysin-rvs — is a key driver of membrane remodeling and fission that counteracts the intrinsic membrane resistance to deformation. This is a key process that enables essential cellular processes, such as the formation of trafficking vesicles, viral egress, and cytokinesis [35]. These domains have been shown to polymerize into cylindrical structures on membrane surfaces and, together with other proteins, like the dynamin fission machinery and the actin cytoskeleton, to potentiate formation of tubular structures and of membrane fission [35]. SNXs containing C-terminal BAR domains play a role in endosomal tubule formation facilitating cargo transport [36], and in membrane deformation during clathrin-coated vesicle formation [37], relying on the membrane curvature sensing abilities of this domain. SNXs-BAR proteins are also important for cargo recognition; where, the sequence-dependent cargo interaction is of significance for the SNX-BAR-mediated biogenesis of tubular profiles. Specifically, the cell couples cargo recognition with the biogenesis of tubular profiles to enable endosome to Golgi transportation routes, and possibly of additional trafficking highways [38]. The cell requirement for such a variety of PX-BAR domain containing SNXs remains an open question, with some authors hypothesizing that distinct members might act in different intracellular compartments, relying on specific signaling events or on the interaction with specific protein motifs. Nevertheless, their co-existence in vivo as homo-/hetero-dimers or higher-order oligomers, crucial for their function, renders the clarification of these processes difficult. Clearly, some members share exchangeable roles, as for instance SNX1/SNX2 and SNX5/SNX6 are all involved in retrograde sorting through the retromer complex [39–41] (discussed in further detail in section 2.2) and display similar biochemical properties. Nevertheless, SNX1 and SNX2, or SNX5 and SNX6, also have unique properties and independent functions in sorting, which are not interchangeable, highlighting both the complexity of this subfamily and how much remains to be unrevealed [42–45]. Less studied to date are the remaining SNX-BAR domain-displaying members SNX4, SNX7, SNX8, SNX30, and SNX32. SNX4 seems to play a role in the indirect recycling pathway to the perinuclear endosomal recycling compartment (ERC), namely of the transferrin receptor [46, 47], but also in the endosome to the trans-golgi network (TGN) pathway [48]. Distinct studies highlight SNX4 interaction with proteins important for membrane trafficking, such as amphiphysins (of relevance in the brain for the recruitment of dynamin into sites of clathrin-mediated endocytosis), dynein, clathrin heavy chain, and tubulin [49]. Thus, authors hypothesize that SNX4 links membrane carrier formation to motor driven movement. SNX7 and SNX8 have been recently associated with infection and with brain pathology [50–53]. Their function remains yet to be mechanistically elucidated, particularly concerning BAR domains’ contribution. Interestingly, studies demonstrated that SNX8 co-localizes partially with retromer components [54], possibly through its BAR domain. SNX30 and SNX32 have not been ascribed to play a role in endosome-to-TGN or with recycling, but, of notice, SNX30 displays a ubiquitious expression profile, whereas SNX32, regarded as SNX6B, is brain enriched, being of possible relevance in the context of the (dys)function of the nervous system.

Within the SNX-BAR subfamily, the subgroup SH3-BAR can be identified: SNX9, SNX18, and SNX33 (Fig. 1). These SNXs display a N-terminal SH3 (Src Homology 3) domain, involved in protein-protein interactions, and a linker region between the PX and the SH3 domain that binds clathrin and AP-2 [1]. No consensus has been reached concerning their ability to form homo- or heterodimers. All of its members are involved in clathrin-mediated endocytosis, particularly in fission among membrane deformation processes [2], being also relevant for autophagosome biogenesis and mitosis [55]. SNX9 is the most well-known member and aids in vesicle formation, acting as a scaffold for assembly and recruitment of main vesicle components, like dynamin, clathrin, AP-2, and components of the actin cytoskeleton [37]. SNX18 and SNX33 seem to have similar subcellular localization as SNX9, being co-localized in the same endosomal structures, but simultaneously also having their unique expression profile and distinct functional specificities. All have been demonstrated to bind dynamin and are, as such, suggested to act on dynamin-dependent endocytic processes (similarly to SNX9). Interestingly, both SNX9 and SNX33 have also reported roles in the regulation of amyloid percursor protein (APP) trafficking, which is of relevance to AD, and will be discussed further in this review in section (4.1) [2]. The SH3 domain is, however, also found in two other SNXs — the SNX26 and SNX28 — where, SNX26 is a GTPase-activating protein that appears to be ubiquitously expressed in humans, and SNX28 (or NOXO1) is a NADPH oxidase organizer. These constitute and additional SNX subgroup — the SH3 — and are deprived of an annotated BAR domain (Fig. 1). As above-mentioned, the SH3 domain potentiates protein-protein interactions by binding target proteins with proline-rich motifs, including those associated with the actin cytoskeleton. Both possibly of relevance for intracellular signaling, their function remains nonetheless to be elucidated.

SNX17, SNX27, and SNX31 assemble into the SNX-FERM subfamily (Fig. 1). The FERM (protein 4.1/ezrin/radixin/moesin) domain has been identified in several molecules and plays pivotal roles in lipid-protein interaction, contributing to membrane tethering and to the interaction with cytosolic parts of transmembrane protein cargoes. The FERM domain is composed by three modules: F1, F2, and F3 [56]. These modules are distinct in their fold and function, with F1 displaying an ubiquitin-fold, F2 a α-helical structure and F3 being similar to phosphotyrosine-binding domains. SNX17 and SNX31 display conserved F1 and F3 modules, and an altered F2 module, and display a FERM-like domain [57]. SNX27 has also been assigned to this FERM-domain subfamily, since in addition to a PDZ domain (of relevance to the nervous system function) it also displays a Ras domain that is highly similar to the F1 module [57]. All members of this family share the ability to bind to proline rich sequences, namely to Asn-Pro-Xaa-Tyr (NPxY) motif-containing cargoes, and associate with H-Ras. This subfamily plays roles both in endosomal trafficking and in signaling processes, highlighting this subfamily ability to couple receptor trafficking and signaling outcomes. From the 3 members that compose the SNX-FERM subfamily, SNX27 is the best characterized, being extensively studied in the context of the CNS homeostasis regulation. SNX27 is important for higher-order processes, such as learning and memory [16], which will be discussed in more detail in section (3). Interestingly, SNX27 is brain-enriched, and is the only SNX that displays a PDZ domain (PSD-95, Disc-large and ZO-1). Although distinct studies have identified numerous SNX27 interacting cargoes [58], it was first identified as a molecule upregulated upon stimulation of dopamine receptors with metamphetamines. In contrast, SNX17 has been shown to regulate the intracellular sorting of the low-density lipoprotein receptor (LRP1) [59], P-selectin [60] and also APP [13], influencing mainly their endocytosis and recycling from/to the cell surface. Interestingly, SNX17 also interacts with numerous SH3-domain containing proteins through its PX domain. In another note, SNX31 is an urothelium-specific (enriched) SNX, reported to play a role in the degradative sorting of uroplakins [61] and associated with integrin degradation and stability [62]. Interestingly, although its expression in the brain seems negligible (NCBI, EST profile data), SNX31 was regarded as a genetic risk factor for the onset of schizophrenia [63]. If this is due to its expression in the nervous system, or other systems, remains to be elucidated.

Finally, the SNX-PXA-RGS-PXC subfamily displays a regulator of G-protein coupled signaling domain (RGS) (Fig. 1). This family is formed by SNX13, SNX14, SNX19, and SNX25. The RGS domain plays important roles in the attenuation of G-protein coupled receptors and related G-protein signaling and, consequently, its members (excluding SNX19 which is deprived of a RGS domain) are regarded as negative regulators of G-protein related signaling cascades. These SNXs also display a N-terminal (PXA) and C-terminal (PXC) PX-associated domains, whose function remains to be elucidated. All members of this family present putative transmembrane domains on their N-terminals. Regarding phosphoinositide (PIP) affinities, SNX13 and SNX19 have been shown to bind preferentially to PI3P, whereas SNX14 and SNX25 display altered binding pockets that do not seem to specifically bind to PI3P [27], which supports a broader PIP binding capability of SNXs-PX domains and their ability of recruitment to a wider array of organelles and membrane domains (or microdomains). Function wise, SNX13 is crucial for mouse development [64], with null-embryos displaying impairments in nutrient uptake and transport, and also aberrant endosome morphology, highlighting this subfamily role both in signaling and trafficking. Of notice, in adulthood, SNX13 is virtually absent in the brain [65]. SNX13 also seems relevant for cardiac function [66], since its reduction is associated with heart failure. On another hand, SNX14 was shown to be of significant relevance for the nervous system homeostasis, being maternally imprinted and crucial for neuronal excitability and synaptic transmission [23]. SNX19 role is still quite poorly explored; nevertheless, available data associates it as genetic risk factor for schizophrenia [18–20, 50]. Finally, SNX25 has been shown to attenuate TGF-β signaling by regulating the lysosomal degradation of the TGF-β receptor [67], and more recently to be of relevance for TrkB receptor degradation [68], rendering it feasible an association between SNX25 with BDNF-TrkB signaling modulation. SNX25 expression was initially identified in neurons and astrocytes [67], but recently using immunohistochemistry and in situ hybridization SNX25 expression was shown to be widespread in neurons but not on GFAP-positive astrocytes, with the exception of the Bergmann glia in the cerebellum [68].

The remaining SNXs are either those containing a PXB-associated domain (SNX20 and SNX21), or other sequences whose structure/domains remain to be characterized or even assigned into a subfamily — such as, SNX15, SNX16, SNX23, SNX29, and SNX34 (Fig. 1). Most are poorly characterized (or of unknown function); but, nonetheless, some have been associated with nervous system homeostasis such as SNX15, SNX16, and SNX21. SNX15, for instance, accelerates APP recycling back to the cell surface and, thereby, reduces amyloid βeta (Aβ) production [69]. Accordingly, SNX15 overexpression in an AD mouse model reduces hippocampal Aβ levels, improving memory [69]. SNX16 seems to regulate pre-synaptic trafficking events, promoting synaptic growth signaling by interacting with the nervous wreck (Nwk) protein in *Drosophila melanogaster* [70, 71]. SNX16 also regulates the tubulation and distribution of neuronal endosomes [72]. SNX21 was recently linked to Huntingtin’s trafficking, which could become relevant to further comprehend Huntington’s disease [73]. SNX20, the other PXB-domain containing SNX, remains to be functionally characterized, although some evidence suggest that it might regulate P-selectin glycoprotein ligand 1 (PSGL-1) [74]. Interestingly, SNX20 gene *locus* has been recently proposed as of relevance to inflammatory bowl’s disease [75]. Data implies that although both display similar domain organization and scaffold ability of their PXB domains, SNX21 N-terminal is quite distinct from SNX20's [76], which could justify differential cargo binding.

Overall, it is clear that domain diversity surpasses the unifying presence of a PX domain within the sorting nexin family, highlighting its significance and their implications in a wide range of processes, which are far beyond the simple ability of binding PIPs within endocytic organelles. How SNXs orchestrate the interaction within their domains at the “intra” and “inter” levels, and how they function (and cooperate) remain open questions. Clearly, the cell relies on numerous trafficking mechanisms and in the collaboration between distinct SNXs members to regulate its plasma membrane component and that of its distinct endocytic organelles. Whether these interactions are physiological or circumstantial is still debatable.

### SNXs and Cellular Sorting Complexes — Contributions from the Retromer, Retriever, CCC and WASH-Complex in Cargo Sorting

**Fig. 2:**
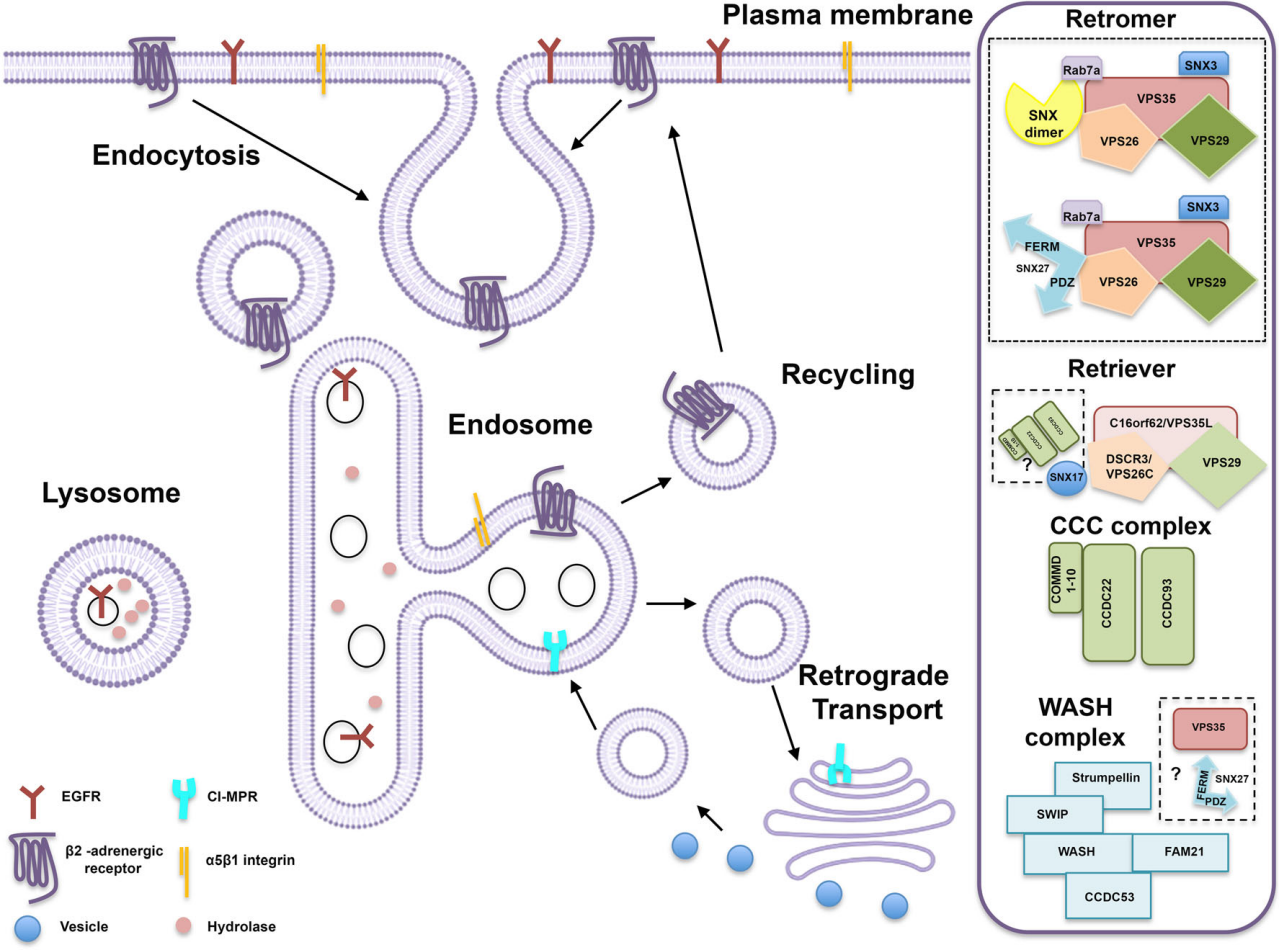
Overview of cellular sorting complexes for cargo retrieval and/or recycling. Schematic representation of the endolysosomal system sorting distinct cargoes from the PM such as the β2-adrenergic receptor, the epidermal growth factor receptor and α1β5-integrin, or those from the biosynthetic pathway such as the cation-independent mannose 6-phosphate receptor (CI-MPR). Representation of the distinct and evolutionarily conserved sorting complexes is depicted, as well as the known SNXs involved and possible adaptors for membrane association. In summary, different protein cargoes are internalized by endocytosis and translocated to early endosomes that also accepts cargoes from the Trans-Golgi network (TGN). The nature of the cargoes that enters the endosome will then dictate their final destination. For instance, neurotransmitter and nutrient receptors will be recycled back to the PM through a direct (fast) or indirect (slower) pathway (β2-adrenergic receptor), whereas receptors for lysosomal enzymes will be sorted by the retrograde pathway to the TGN (CI-MPR). Ubiquitylated membrane proteins in turn will be sorted into intraluminal vesicles (ILVs) and degraded in the lysosome (EGFR). Thus, protein cargoes are either degraded or retrieved from the degradative pathway being either recycled to the cell surface, to the TGN or to other organelles. Distinct ancient and conserved retrieval complexes are displayed on the right side of the figure: retromer complex (and SNX combinations); the retriever; the CCC complex and the WASH complex. These complexes are spatially segregated from the degradative sub-domains within the endosome, those containing endosomal sorting complex required for transport (ESCRT). Retromer complex and the retromer-associated proteins SNX3 and Rab7a (that facilitate retromer recruitment to the endosomal membrane) are responsible for the retrograde sorting of several cargo proteins either to the PM or to the TGN, namely of the β2-adrenergic receptor and CI-MPR. The CRC is composed by VPS26, VPS29, and VPS35 that recognizes cargo and the SNX dimer provides membrane association and deformation. SNX27, functions as a cargo adaptor establishing associations through its PDZ domain (with VPS26) and FERM domain (with FAM21) to prevent PDZ-containing cargoes to be sorted to the lysosome for degradation or to the TGN. Some cargoes such as the Wntless receptor require SNX3 for retrograde sorting and do not depend on SNX dimer or SNX27 sorting. The WASH complex aids in actin polymerization driving the formation of actin-enriched niches that confine protein cargoes, promoting the biogenesis of tubular structures and/or vesicles that pursue with cargo recycling. The COMMD/CCDC22/CCDC93 (CCC) complex is also involved in the regulation of membrane protein recycling and is important for retriever complex recruitment. The retriever complex also shapes cargo retrieval and recycling, preventing the degradation of bout 120 cargoes including β-integrin, and relies on SNX17 as a cargo adaptor. Its membrane recruitment is retromer-independent. CCDC, coiled-coil domain containing; CI-MPR, cation-independent mannose 6-phosphate receptor; COMMD, copper metabolism MURR1 domain-containing; CRC, cargo recognition complex; DSCR3, Downs syndrome critical region 3; EGFR, epidermal growth factor receptor; FAM21, family with sequence similarity 21; SWIP, strumpellin and WASH interacting protein; VPS35, vacuolar protein sorting protein 35; VPS35L, vacuolar protein sorting protein 35-like. Figure was adapted from [77]

SNXs family members form heterogeneous protein complexes with other proteins to achieve proper protein-cargo sorting within the endolysosomal system (Fig. 2). These complexes play active roles in sequestering protein cargoes from the degradative lysosomal pathway. In this context, the best-studied protein complex is the retromer complex, which aids in the retrieval and recycling of several cargoes away from the endolysosomal pathway [7]. Conserved throughout evolution, this heteropentameric complex is composed in mammals by the cargo recognition complex (CRC) formed by Vps26 (isoforms A and B — involved in cargo specificity), Vps29 and Vps35, as in yeast, and by a dimer of SNX1, SNX2, SNX5, and SNX6 [78]. Complex recruitment to endosomal membranes is debatable but proposed to occur through SNX’s ability to bind PI3P and by Vps35 interaction with the Rab7 effector protein [79]. Aiding in this process, the ends of CRC trimer Vps26, VPS29, and Vps35 are responsible for binding SNX-BAR N-terminals [80]. Interestingly, association of Vps trimer with Rab7a is also highly conserved throughout evolution [81]. Of notice, SNX3 and SNX27 have both been demonstrated to sort cargoes through the retromer complex, although lacking membrane-sensing BAR-domain, adding layers of complexity to the retrograde-trafficking [28, 82–85]. A distinct and evolutionarily conserved retromer complex was in this manner shown to exist, independently of SNX-BAR-retromer containing heterodimers (Fig. 2) [28]. Of notice, we have highlighted neuronal-dependent phenotypes that require SNX3 function independent of retromer components, and that, thus, SNX3 is also involved in retromer-independent mechanisms [24, 29, 32]. Interestingly, in mammalian cells, both SNX3- and SNX-BAR retromer complexes are segregated, being spatially separated along the endolysosomal pathway [28]. SNX3 is recruited to early endosomes, while the SNX-BAR retromer complex is more abundant in latter stages of endosomal maturation (early-to-late endosome) [28, 49, 86]. Possibly cargoes can either initially enter the SNX3-retromer sorting pathway or proceed to the SNX-BAR retromer to be recycled or sorted to the TGN. They may also, ultimately, be sorted into intraluminal vesicles (ILVS) and thereafter for lysosomal degradation. Furthermore, SNX27 can also serve as cargo specific adaptor to the SNX-BAR retromer complex, being involved in the sorting of numerous receptors [82] (Fig. 2). Supposedly, other SNXs might serve as cargo adaptors to promote retrograde sorting which remains to be elucidated. Moreover, SNX-BAR retromer itself can also selectively interact with different cargoes [87].

Complexity of endolysosomal cargo sorting does not end with retromer cargo diversity and the range of distinct adaptors and retromer components. SNX-BAR retromer complex interactions have been identified with other complexes, such as the macromolecular WASH complex [88], the retriever [89], and the COMMD/CCDC22/CCDC93 (CCC) complexes [77, 90] (Fig. 2). The WASH complex is formed by a nucleation-promoting factor, the Wiskott-Aldrich syndrome homologue 1 (Wash1, that localizes to endosomes, and by Fam21, strumpellin, CCDC53 and KIAA1033 [91]. Authors have shown that endosomal localization of WASH complex relies on its interaction with CRC through Vps35 [88], but not exclusively. In this manner, retromer can regulate cargo-sequestering by recruiting WASH complex to trigger actin polymerization and, consequently, drive formation of actin-enriched niches to confine protein cargoes, aiding in the biogenesis of tubular structures and/or vesicles that pursue with cargo recycling. Thereby, WASH depletion leads to defects in general cargo sorting, impacting on cargo recycling, endosome-to-Golgi and endosome-to-lysosomal trafficking. Curiously, FAM21, a WASH complex component, was recently shown to direct SNX27-retromer cargoes to the plasma membrane, preventing its transport to the Golgi apparatus (TGN pathway) and lysosomes [92]. This highlights the significance of the interactions established by the trafficking machinery (endosomal coats) with the action cytoskeleton, so to instigate the generation of trafficking vesicles and to shape trafficking routes.

The fact that several cargoes, such as β-integrin, do not rely on the retromer for their recycling to the cell surface, suggests that other transportation “highways” exist within the cell that are retromer-independent. Recently, Cullen and collaborators showed the existence of the retriever complex — a complex that associates with SNX17 and CCC and WASH complexes to promote recycling of β-integrin and, in this manner, prevent its lysosomal degradation [89] as well as that of other 120 cargoes [89]. Like the retromer, the retriever complex is also an ancient and conserved multiprotein complex that shapes cargo retrieval and recycling, preventing cargo degradation. It is composed by DSCR3, C16orf62, and by Vps29, an arrestin-like protein that also forms the retromer complex [89]. Interestingly, both retromer and retriever associate with selective-cargo adaptors, the SNX27 and SNX17 (Fig. 2), respectively, providing specificity for cargo engagement within these retrieval pathways. Both complexes were shown to reside on endosomal membranes and to be segregated from the degradative subdomain (ESCRT-enriched). Recruitment of retriever is independent of SNX3 or Rab7 and, thus, retromer-independent [89]. Another complex of relevance is the COMMD/CCDC22/CCDC93 (CCC) complex, also involved in the regulation of membrane protein recycling [90]. Interestingly, the CCC complex is important for retriever complex recruitment, since its suppression was shown to perturb endosomal localization of retriever components, but not those of the retromer complex [89]. Furthermore, CCC association with WASH complex component FAM21 is required for its endosomal association [90], and FAM21 is recruited to endosomes both by both retromer-dependent [93, 94] and independent mechanisms [77, 89].

In all, this is indicative that the cell engages in a complex mechanistic molecular loop to synchronize all retrograde-involved sorting complexes and provide cargo-specificity regulation (Fig. 2). Ultimately, it is intriguing to observe that the cell requires several protein complexes, all ancient and evolutionarily conserved, to orchestrate cargo endosomal recycling. Of notice that to do so, the cell relies on the function of distinct SNXs, whether containing or not a membrane curvature sensing domain — BAR domain (like the BAR-deprived SNX3, SNX17, and SNX27). This complexity within endosomal sorting opens the possibility of involvement of other SNXs as potential coat components of the endosomal system, and panoply of protein interactions with other sorting adaptors and effectors. Undoubtedly, by enhancing molecular understanding of this fundamental process within eukaryotic cells, we will shed light on the association between endosomal sorting and human diseases, particularly in those afflicting the nervous system.

### Phylogenetic Analysis of SNXs Across Phyla

The SNX protein family is conserved across phyla, with its members encoded in the genomes of unicellular organisms (that lack nerve cells) to the genome of more complex mammals with a brain [1]. Association of SNXs with the regulation of nervous system homeostasis in mammals underscores the need to pursuit the origins of this family and how their increasing evolutionary complexity parallels the development of the nervous system throughout speciation, in a quest to further understand this association. This issue is *per se* both pertinent and controversial as the availability of an increasing number of decoded genomes has added layers of complexity. We have surveyed evolutionary related literature [95, 96], and opted to include in our phylogenetic analysis the genomes of both prokaryotic and eukaryotic organisms, including in the latter both unicellular and multicellular, and preceding the appearance of nerve cells, hence ranging from protists, fungi, plants, and invertebrates to vertebrates. The first nerve cells evolved after the considered “basal” metazoans — Porifera and Placazoa taxa — that do not display clearly defined neuronal cell types. This notion highlights that the more ancestral metazoans did not bear neuronal cells, and that only more evolved metazoans, like the cnidarian ctenophores and bilateria ancestors, gained the advantage of multisensorial integration coupled with rapid intracellular communication, even over considerable distances [95, 96].

**Fig. 3:**
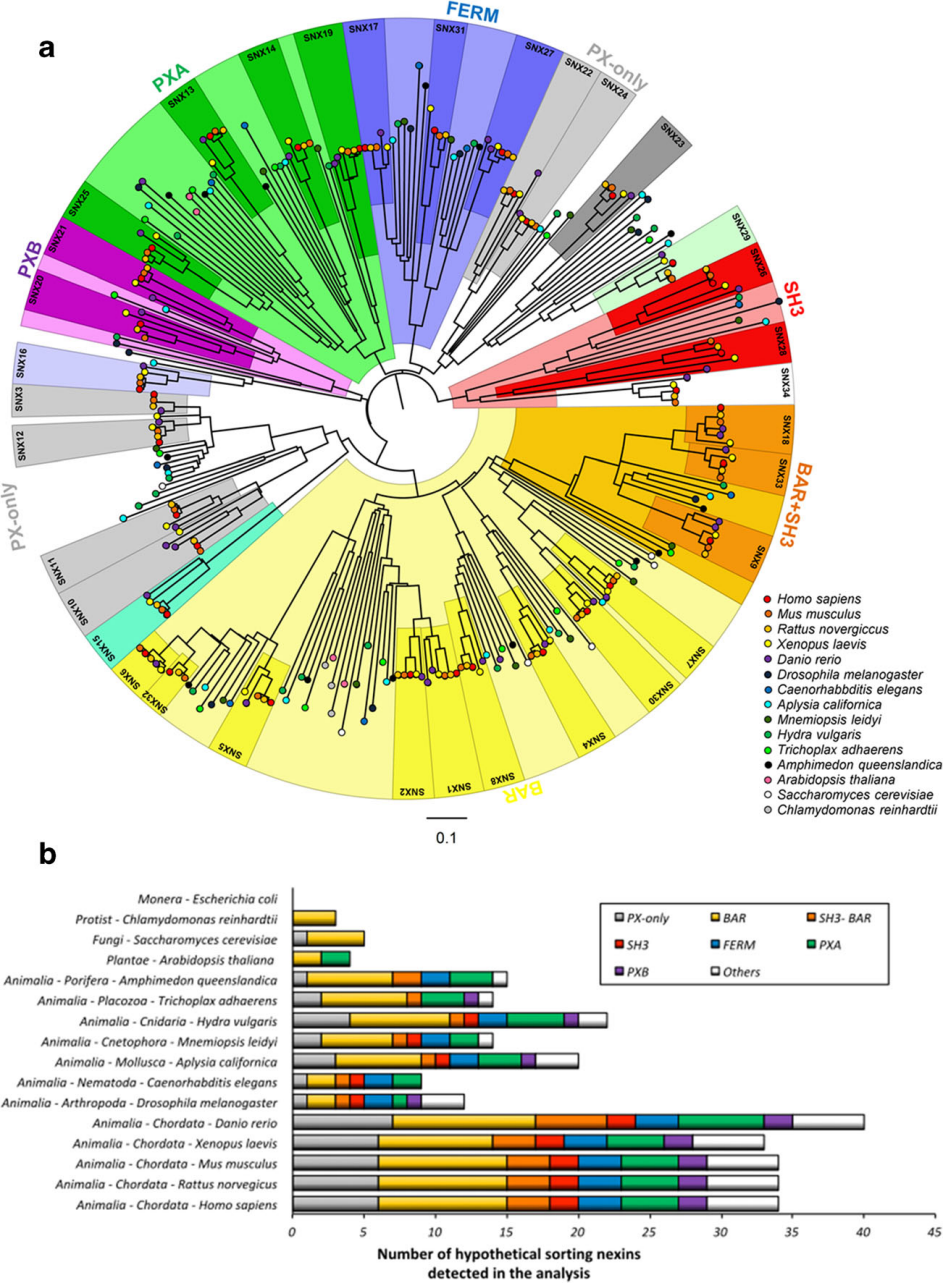
Phylogenetic analysis of SNXs orthologs across phyla, from prokaryotes to eukaryotes, spanning the emergence of nerve cells/nervous system. **a** Unrooted circular tree representing the phylogenetic relationship between SNXs orthologs across different species. Corresponding 293 PX domain containing sequences were aligned using CLUSTALW within MEGA7. MEGA7 was used to reconstruct the phylogeny of SNXs using maximum likelihood with Gamma distributed rates (5 categories). 500 replications were performed for a bootstrap analysis. FigTree was used to display the obtained phylogeny and to color the different clades. Orthologs in *Danio rerio*, *Xenopus laevis*, *Mus musculus*, *Rattus norvegicus*, and *Homo sapiens* were used to indicate the clades containing the different human SNXs (SNX1-34). **b** Distribution and quantification of the different SNXs orthologs within each SNX-subfamilies, across the analyzed species. Phylogenetic analysis and the data obtained with conserved domain analysis (CDD and prosite) were used to characterize SNXs distribution across the representative genomes. SNXs subfamilies were divided in SH3+BAR, BAR, SH3, PXA, PXB, FERM, the ones containing only the PX domain, and others. Species accession numbers and sequences are listed on supplementary material (Table 1-2)

In order to infer phylogenetic relationships between SNXs across phyla, and to survey domain complexity throughout evolution, we conducted a full search, implementing an unbiased procedure, for the presence of sequences compatible with sorting nexins (SNX) proteins signatures, in the genomes of the studied organisms. For this, we downloaded the complete proteome of the representative genome of 15 model organisms in NCBI and an extra one (*Mnemiopsis leidyi*), not available in NCBI, from an exterior database (https://research.nhgri.nih.gov/mnemiopsis/). The list of organisms is displayed in the supplementary information that also includes the methodology and sequences used. Phylogenetic analysis was performed only on variation of the PX domain sequence. However, obtained data indicated that clusters show several monophyletic clades corresponding to the known SNX-associated domains: monophyletic clades exist corresponding to SNXs with the extra BAR, PXB, PXA-RGS, FERM, and SH3+BAR (within the BAR clade) domains. This shows that these SNXs are, as expected, evolutionarily related. SNX sequences that contain only the PX domain are present in two clades — one with SNX23 and SNX24, and a larger clade containing SNX3, SNX10, SNX11, SNX12, and SNX16 — with several related sequences being present in less evolved organisms. SNXs with the SH3 domain (no BAR) are both clustered together and with SNX34 (an SNX with little characterization). These deep phylogenetic relationships show relatively low values of confidence in the bootstrap analysis (15–25%, not shown) but given the millions of years involved in the evolution of these sequences, and the fact that the analysis is solely based on a stretch of about 100 residues, the results are remarkable (Fig. 3a). Interestingly, BAR containing SNXs seem to be more prevalent throughout evolution, possibly due to the role of this domain in membrane remodeling, curvature sensing, and retrograde trafficking through the retromer complex, which has been highlighted across phyla from yeasts to mammals. Of notice, lower organisms display a lower number of BAR-containing SNXs. Possibly throughout speciation, there was a need to diversify the array of available BAR-domain containing SNXs to accommodate more complex molecular processes and to provide higher cargo specificity. The same phenomenon is observed in other clades, just not so markedly evident. Interestingly, the Vps components of the retromer complex, Vps26, Vps29, and Vps35, are also extremely conserved throughout eukaryote evolution [97]. This remarkable conservation of retromer complex, and of the BAR-containing SNXs within eukaryotes, supports retromer as a major deforming complex of relevance to the eukaryote trafficking system [98, 99].

Phylogenetic and conserved domain analyses (CDD and prosite) were used to characterize distribution of different SNXs across representative genomes. Sorting nexins were divided according to their domains: SH3+BAR, BAR, SH3, PXA-RGS, PXB, FERM, the ones containing only the PX domain and others (containing the human SNX15, SNX16, SNX23, SNX29, and SNX34) (Fig. 3b). It is noteworthy that, as anticipated, no SNX-related sequences were found in the bacteria *Escherichia coli* genome and that in all analyzed Eukaryotic genomes at least SNXs containing a BAR domain were detected. This highlights the importance of membrane deformation/fission in endosomal trafficking, and also of retrograde sorting, not only in more complex organisms but also at the unicellular level. Evolutionarily, it is interesting to observe that in organisms such as protists, only BAR-containing SNXs are encoded in their genome, whereas plants also display PXA-containing SNXs (of unknown function). In fungi, PX-only containing SNXs also emerge, which is indicative of the need for BAR-domain deprived sorting, possibly to orchestrate trafficking events through other mechanisms deprived of tubular sorting and membrane fission, or of the recruitment of the above-mentioned retromer complex. Additionally, when transitioning from these lower eukaryote genomes to animals’ genomes, there is an evident increase in the number of SNXs identified and in their domain complexity. This is of relevance when assuming that the first nerve cell-like emerged in the Filo Porifera (*Amphimedon queenslandica*)/Placazoa (*Trichoplax adhaerens*) and the nervous system in the Fili Cnidarians (*Hydra vulgaris*)/Cnetophores (*Mnemiopsis leidyi*) [95, 96]. Nevertheless, while considering the absolute number of orthologs present in *Caenorhabditis elegans* and *Drosophila melanogaster*, we see an evident decrease in SNXs absolute numbers, going against the general evolutionary trend of a higher number of SNXs occurring with the increasing complexity of the nervous system. However, it is important to note that these reductions are mostly caused by lower numbers of SNX orthologs within the BAR and PXA domains-containing SNXs, whose orthologs are expected to substantially overlap in function (as shown in Table 1). Indeed, these two species show an increment in the number of SNX subfamilies in relation to the representative species with less complex nervous systems (or without nervous system), namely the SH3 and FERM in *C. elegans* and the SH3, FERM, and PXB in *D. melanogaster*, displaying thus a higher complexity. Indeed, *D. melanogaster*, often used as a model organism for inferences in humans, shows the same number of subfamilies of SNXs as *Homo sapiens*. More interestingly, a second visible increase is also notorious in the Filo Chordata. Given this, *Danio rerio*, *Xenopus laevis*, *Mus musculus*, *Rattus norvegicus*, and *Homo sapiens* display the higher number of SNXs (possibly corresponding/supporting to a higher nervous system complexity). It is worth pointing out the higher number of detected SNXs in zebra fish (*Danio rerio*). Chromosome duplication occurred deep in the evolution of teleost fish with previous estimates that about 20% of the genes in Zebra Fish have orthologous with millions of years of evolution separating them [155]. This is visible in several SNXs with duplicates in SNX1, SNX8, SNX9, SNX10, SNX18, SNX19, and SNX27. Given this, the higher number of SNXs in zebra fish does not correspond to an increase in diversity of existing SNXs, but rather duplicates of existing SNXs (although clearly differently between them). Looking with more detail into the domains that emerge in this higher order Eukaryotes, it is noteworthy to mention that the FERM domain emerges only in animals but it is absent in the *Trichoplax adhaerens*, a Placazoa deprived of a nervous system. This is interesting since Placazoa indeed possessed nerve-like cells, which were lost throughout evolution, but not a true nervous system [95, 96]. Another interesting finding is that the FERM domain was duplicated in the genome of the ancestral of animals, evolving from there in duplicate, where one gene presented the FERM domain alone (SNX17) and the other gene also displayed a PDZ domain (SNX27).

**Table 1:** Summary of SNXs reported role in the context of nervous system function and/or pathology. In bold are keywords that hihglight SNXs association with nervous system function/homeostasis and with pathological events

	Subfamily	Chromosome (Hs)	EST profile	Function	Notes	References
**SNX1**	PX-BAR	**15;** 15q22.31	**Ubiquitous**	**- Single-nucleotide polymorphism (SNPs)** in brains of **Alzheimer’s** (**AD**) patients; - important for **neurite** outgrowth regulation; - Plays critical role in the trafficking of **mGluR1** in hippocampal neurons;	*H. sapiens* form is highly expressed in the thyroid; Important for retromer-dependent sorting; Mammalian SNX1 is the original family member, discovered on an yeast two-hybrid screen.	[3, 15, 36, 43, 100]
**SNX2**	PX-BAR	**5;** 5q23	**Ubiquitous**	- **Expression is increased 5x** in the hypothalamus of **aging mice**; - Possible role in **epilepsy**; - Associated with **schizophrenia**; - **Expression is decreased** in brains of **AD patients**; - Might play a role in **brain development,** regulating the action of trophic factors;	*H. sapiens* form is ubiquitously expressed; Important for retromer-dependent sorting; Has a C-terminal BAR domain, involved in membrane tubulation.	[50, 101–103]
**SNX3**	PX-only	**6;** 6q21	**Ubiquitous**	**- SNPs** were identified in brains of **AD** patients; - **SNX3 gene is disrupted** in patients with microcephaly, microphthalmia, ectrodactyly, and prognathism (**MMEP**) and mental retardation**; - Expressed in microglia; -** Identified in a Synaptic ubiquitination brain screen**;** **- Enriched** in the **postsynaptic density;** - Important for **neurite outgrowth;** **-** Plays a role in **neurogenesis;** **- Phylogenetically - functionally** related to **SNX12**; - Involved in **Amyloid-beta production**, and **APP internalization**; - **Increased expression** in brains of patients with **schizophrenia;** **- Important** for nematode **neuronal development** and **wiring,** and for **(neuro)behavioral function;** - Important for iron homeostasis in **dopaminergic neurons** which can be of relevance to **PD pathology**; - Important for neural tube closure;	*H. sapiens* form is highly expressed in adrenals, fat and placenta; Important for retromer-dependent sorting of specific cargoes; SNX3 has been annotated as having no identified domains outside of the PX domain.	[15, 24, 104–111]
**SNX4**	PX-BAR	**3;** 3q21.2	**Ubiquitous**	**- Interacts** with **amphiphysins** (important in the brain for recruitment of dynamin to the sites of clathrin-mediated endocytosis); **-** Identified in a Synaptic ubiquitome brain screen; **- Interacts** with **BACE1 regulating amyloidogenic processing;**	*H. sapiens* form is highly expressed in the kidney and thyroid; Has a C-terminal BAR domain, involved in membrane tubulation.	[106, 112]
**SNX5**	PX-BAR	**20;** 20p11	**Ubiquitous**	**-** No clear association with the nervous system function; - Association with **Dopamine receptor 1 trafficking**;	*H. sapiens* form is highly expressed in the thyroid; Important for retromer-dependent sorting; Has a C-terminal BAR domain, involved in membrane tubulation.	[113]
**SNX6**	PX-BAR	14; 14q13.1	**Ubiquitous**	- **Decreased** expression in brains of AD patients; - Interacts with **BACE1** and mediates APP processing/Aβ generation **(negative regulator);** **- modulated by miR-98-5p - proposed target for AD;** **-** Associated with **schizophrenia**; - **Ablation** leads to defects in **synaptic function** of CA1 pyramidal neurons and spatial memory;	*H. sapiens* form is ubiquitously expressed; Important for retromer-dependent sorting; Has a C-terminal BAR domain, involved in membrane tubulation.	[12, 50, 114–117]
**SNX7**	PX-BAR	1; 1p21.3	**Ubiquitous**	**-** Associated with **Aβ production**, by regulating **APP lysosomal degradation**; - Controls the activity of the **kynurenine pathway**, which plays important roles in **schizophrenia** and **bipolar disorders**;	*H. sapiens* form is highly expressed in the colon and reduced in the brain; Has a BAR domain, involved in membrane tubulation.	[51, 52]
**SNX8**	PX-BAR	7; 7p22.3	**Not Ubiquitous**	- Associated with schizophrenia; - Enhancer for **Aβ toxicity;** **- SNPs** have been associated with the risk of late onset **AD;** - Attenuates **Aβ accumulation** and **memory deficits in AD** mouse model, by enhancing **non-amyloidogenic APP trafficking;** **-** Important for **neurodevelopment;**	*H. sapiens* form is highly expressed in the spleen; Has a C-terminal BAR domain, involved in membrane tubulation.	[50, 53, 118, 119]
**SNX9**	SH3-PX-BAR	6;6q25.1-q26	**Ubiquitous**	**- Regulates APP processing/Aβ generation;** - Present in **presynaptic** vesicles; - Regulates **synaptic vesicles** endocytosis; - Levels in cultured **neurons** are decreased by **alcohol exposure**; - **Adaptor** for **synaptojanin-1**, a key player in synaptic vesicle recovery at the synapse; - Not essential for mice developing and hearing;	*H. sapiens* form is ubiquitously expressed; In addition to the BAR domain, it also displays a N-terminal SH3 protein-interacting motif, reported to bind to polyproline and hydrophobic aminoacidic sequences.	[14, 120–123]
**SNX10**	PX-only	7; 7p15.2	**Ubiquitous**	- Genetic marker of **longevity** in the **Amyotrophic Lateral Sclerosis** SOD1(G93A) mouse model; - Highly expressed in dopaminergic neurons;	*H. sapiens* form is enriched in the brain; SNX10 has been annotated as having no identified domains outside of the PX domain.	[124, 125]
**SNX11**	PX-only	17q21.32	**Ubiquitous**	**-** Possibly involved in thermosensing;	*H. sapiens* form is ubiquitously expressed; SNX11 has been annotated as having no identified domains outside of the PX domain.	[126]
**SNX12**	PX-only	**X;** Xq13.1	**Ubiquitous**	**- Decreased expression** in the brains of **Alzheimer’s** (AD) patients**;** **- Interacts** with **BACE1 and mediates APP processing/Aβ** generation; - Regulates neurite formation**;** **-** Plays a role in **neurogenesis;** **-** Identified in a **Synaptic ubiquitination rat brain screen;** **- Aging biomarker** correlated with **AD;**	*H. sapiens* form is ubiquitously expressed; SNX12 has been annotated as having no identified domains outside of the PX domain.	[12, 127]
**SNX13**	PXA-RGS-PX-PXC	7; 7p21.1	**Ubiquitous**	**-** No clear association with the nervous system function; - Crucial for mouse development; namely for neural tube closure;	*H. sapiens* form is ubiquitously expressed; Displays a PX-associated domain A, expected to bind fatty acids; a PX-associated domain C, of unknown function; two N-terminal hydrophobic domains expected to form transmembrane helices. It also displays a RGS domain, that activates GTPase activity of heterotrimeric G α-subunits; In this manner, it is expected to shut-off G protein-coupled receptor signaling pathways.	[64]
**SNX14**	PXA-RGS-PX-PXC	6; 6q14.3	**Ubiquitous**	- Expressed in motoneurons and **upregulated** in the presence of **lithium;** **- Regulates neuronal excitability** and promotes **synaptic transmission;** **- Mutations in SNX14** cause **cerebellar ataxia** and **Intellectual disability syndrome;** **- SNX14 mutations** affect neutral **lipid metabolism** in autosomal recessive **spinocerebellar ataxia 20;** **- Associated** with **cerebellar ataxia disease;**	*H. sapiens* form is enriched in testis; Displays a PX-associated domain A, expected to bind fatty acids; a PX-associated domain C, of unknown function; two N-terminal hydrophobic domains expected to form transmembrane helices. It also displays a RGS domain, that activates GTPase activity of heterotrimeric G α-subunits; In this manner, it is expected to shut-off G protein-coupled receptor signaling pathways.	[11, 23, 65, 128–131]
**SNX15**	PX-MIT	11; 11q12	**Ubiquitous**	- Promotes **APP** recycling to the cell surface and Aβ generation;	*H. sapiens* form is ubiquitously expressed; SNX15 includes a C-terminal microtubule interacting and trafficking (MIT) domain, expected to contribute to membrane association.	[69]
**SNX16**	PX-SNX16	8; 8q21.13	**Ubiquitous**	- Interacts **with Nwk (Nervous Wreck)** ***D. melanogaster*** **in a pre-synaptic manner;** **-** Regulates **presynaptic trafficking;** **-** Promotes **synaptic growth factor signaling;** **- Controls** tubulation and distribution of **neuronal endosomes;**	*H. sapiens* form is ubiquitously expressed; SNX16 possesses significant predicted helical structure from between approximately residues 220 and 310 downstream of the PX domain.	[70–72]
**SNX17**	PX-FERM	**2;**2p23.3	**Ubiquitous**	**- Regulates APP** processing and **Aβ generation;** **-** Endocytosis and recycling of **LDLR family members, LDLR, VLDLR, ApoER2 and LRP1;** **- Interacts** with the **cerebral cavernous malformation related protein KRIT1;** **- Upregulated in PFC of mice treated with Ethanol;** - Associated with **schizophrenia**; **- SNX17** is important for cell fate determination and **neurogenesis;**	*H. sapiens* form is ubiquitously expressed; Important for retromer-dependent sorting; Important for retriever-dependent sorting; Displays a FERM domain, a C-terminal 4.1, ezrin, radixin, moesin protein-protein interacting domain; PX-FERM-domain containing proteins are expected to function as hubs for endosomal trafficking, and Ras-mediated signaling.	[13, 50, 132–134]
**SNX18**	SH3-PX-BAR	**5;** 5q11.2	**Ubiquitous**	**- Dynamically** regulated in **developing spinal motor neurons;** **- Expressed in dorsal root ganglia neurons;** **-** Might play a role in **axonal elongation;** - Associated with **schizophrenia**;	*H. sapiens* form is enriched in the bone marrow; In addition to the BAR domain, it also displays a N-terminal SH3 protein-interacting motif, reported to bind to polyproline and hydrophobic aminoacidic sequences.	[50, 135]
**SNX19**	PXA-RGS-PX-PXC	**11;**11q24.3-q25	**Ubiquitous**	**-** Involved in **schizophrenia**	*H. sapiens* form is ubiquitously expressed; Displays a PX-associated domain A, expected to bind fatty acids; a PX-associated domain C, of unknown function; two N-terminal hydrophobic domains expected to form transmembrane helices.	[18–20, 50]
**SNX20**	PX-PXB	**16**;16q12.1	**Lung, lymph node, spleen**	**-** No clear association with the nervous system function;	*H. sapiens* form is enriched in the lungs, lymph nodes and spleen; highly expressed in macrophages and immune cells; SNX20 and SNX21 are highly homologous and share a conserved helical domain; Displays a PXB domain of ∼140 residues downstream of the PX domain.	
**SNX21**	PX-PXB	**20;** 20q13.12	**Ubiquitous**	**- SNX21** is important for **huntingtin recruitment;**	*H. sapiens* form is enriched in the skin, and in fetal liver tissue; SNX20 and SNX21 are highly homologous and share a conserved helical domain; Displays a PXB domain of ∼ 140 residues downstream of the PX domain.	[73]
**SNX22**	PX-only	**15;** 15q22.31	**Ubiquitous**	**-** No clear association with the nervous system function;	*H. sapiens* form is enriched in the thyroid; These proteins have been annotated as having no identified domains outside of the PX domain. In all cases, the proteins possess short extra sequences . Without predicted secondary structure. The C-terminal tail of HS1BP3 is a proline-rich region.	
**SNX23/Kif16B**	Kinesin-PX	**20;**20p12.	**Ubiquitous**	**-** No clear association with the nervous system function;	*H. sapiens* form is enriched in the adrenals; Displays a N-terminal kinesin motor domain, expected to contribute to endosomal transport through the microtubule network; and a putative forkhead association (FHA) domain and a central coiled-coil dimerization domain, that play a role in phosphoprotein binding.	
**SNX24**	PX-only	**5;** 5q23.2	**Ubiquitous**	**-** No clear association with the nervous system function;	*H. sapiens* form is ubiquitously expressed; SNX24 has been annotated as having no identified domains outside of the PX domain.	
**SNX25**	PXA-RGS-PX-PXC	**4;** 4q35.1	**Lung enriched**	**- Localizes to neurons and Bergmann glia**; - **Increased** expression in temporal lobe **epilepsy** patients; **- Regulates TGF-β** signaling by enhancing **receptor degradation;** **-Genetic modifier** of **age** at **Early** and **late** onset of **Alzheimer's disease;** - Might regulate **circadian pacemaking system;** **- Regulates tyrosine kinase receptor B** trafficking**;** possibly modulates **BDNF-TrkB signaling;**	*H. sapiens* form is enriched in the lung	[17, 67, 68, 136, 137]
**SNX26/ARHGAP33**	PX-SH3-GAP	**19;**19q13.12	**Ubiquitous**	**-** Involved in dendritic spine formation of mature neurons; - Essential for synapse development; - Regulator of intracellular trafficking of TrkB, a high-affinity receptor of the brain-derived neurotrophic factor; - important for insulin-stimulated glucose metabolism;	*H. sapiens* form is enriched in the testis; Display a SH3 protein-interacting motif, reported to bind to polyproline and hydrophobic aminoacidic sequences; and a very long C-terminal domain with no annotated structural motifs.	[138–140]
**SNX27**	PX-FERM	**1;** 1q21.3	**Brain enriched**	**- Psychostimulant-induced gene** **- Highly expressed in the brain**: - Interacts with several cargoes: N-Methyl-d-aspartate (NMDA) receptor 2C (NR2C); a G protein gated inwardly rectifying potassium channel and 5-HT4a receptor; - **Snx27**^**-/-**^ mice have **memory/learning deficits and excitatory synaptic dysfunction;** **- Snx27 expression is decreased in Down's syndrome** patients (and mouse model). **SNX27 up-regulation rescues learning defects;** **-** Expression is **decreased in AD patients;** **-** Inhibits amyloidogenic processing by regulating y-secretase; **-** Promotes **APP recycling** to cell surface by interacting with **SorlA;** **- Deletion** promotes recovery from s**pinal cord injury,** by reducing **microglia proliferation;** **- Down-regulation** does not exacerbate **amyloidogenesis in APP/PS1 AD mice;** **- SNX27 induces myoclonic epilepsy:** **- SNX27 variants** are associated with **seizures, developmental delay, behavioral impairments and brain abnormalities;** **- SNX27** regulates **GPR17 trafficking,** being involved in **oligodendrocyte differentiation;** **- Promotes neuroligins sorting,** important for **synaptic transmission and inhibitory signaling;**	*H. sapiens* form is enriched in the testis; Displays a FERM domain, a C-terminal 4.1, ezrin, radixin, moesin protein-protein interacting domain; PX-FERM-domain containing proteins are expected to function as hubs for endosomal trafficking, and Ras-mediated signaling.	[17, 141–152]
**SNX28/NOXO1**	PX-SH3	**16;** 16p13.3	**Not ubiquitous**	**-** No clear association with the nervous system;	*H. sapiens* form is enriched in the colon; Displays C-terminal SH3 domains, reported to bind to polyproline and hydrophobic aminoacidic sequences.	
**SNX29**	SNX29-PX	**16;** 16p13.13-p13.12	**Ubiquitous**	Associated with schizophrenia;	*H. sapiens* form is enriched in the kidney; SNX29 has been annotated as having no identified domains outside of the PX domain; a helical structure has been predicted with significance, upstream of the PX domain.	[50]
**SNX30**	PX-BAR	**9;** 9q32	**Ubiquitous**	**-** No clear association with the nervous system;	*H. sapiens* form is enriched in the kidney and lungs; Has a C-terminal BAR domain, involved in membrane tubulation.	
**SNX31**	PX-FERM	**8;** 8q22.3	**Bladder; and in a minor level in the brain, esophagusprostate and testis**	- Associated with schizophrenia;	*H. sapiens* form is enriched in the bladder; Displays a FERM domain, a C-terminal 4.1, ezrin, radixin, moesin protein-protein interacting domain; PX-FERM-domain containing proteins are expected to function as hubs for endosomal trafficking, and Ras-mediated signaling.	[50]
**SNX32**	PX-BAR	**11;** 11q13.1	**Brain enriched**	**-** Associates with increased risk of **Alzheimer disease;** **- Genetic variants** associate with **neurological phenotypes;**	*H. sapiens* form is enriched in the brain; Has a C-terminal BAR domain, involved in membrane tubulation.	[153]
**SNX33**	SH3-PX-BAR	**15;** 15q24.2	**Ubiquitous**	**- Regulates APP processing/Aβ generation;** **- required for mitosis; interacts with Wiskott-Aldrich syndrome protein;** **- interferes with cellular PrP formation by modulation of PrP shedding;**	*H. sapiens* form is ubiquitously expressed; In addition to the BAR domain, it also displays a N-terminal SH3 protein-interacting motif, reported to bind to polyproline and hydrophobic aminoacidic sequences.	[14, 154]
**SNX34**	PX-SNX34	**6;** 6p25.2	**Ubiquitous**	**-** No clear association with the nervous system function;	*H. sapiens* form is ubiquitously expressed; SNX34 was recently identified by bioinformatics searches.	[2]

Why would the nervous system, and more precisely neurons, require the emergence of specific domains within the SNX family, is still an open question. It also remains to be explored if the requirements for these domains are also species-specific, or if these SNXs vary at the expression level among species. It is intriguing to hypothesize that these domains play important roles within neuronal cells (or other cells of the nervous system, namely glia), possibly emerging from the specificities of cell populations. Clearly, throughout speciation, neural cells increased significantly in number but also in the diversity of neuron subtypes within the nervous system itself, rendering neurons the most probable diverse cell type [95, 96]. At the molecular level, it is still intriguing to distinguish between a “regular” cell and a neuronal cell, since both share similar machineries to perform vital functions for cell survival. Nevertheless, neurons are quite distinct from other cell types in what concerns communication, being unique on how they establish synaptic connections and emit electrochemical signals. Traffic wise, some adaptations also seem to have taken place throughout evolution to accommodate the demands of a highly polarized cell, with tremendously long extensions, and the demand for fast protein turnover at the synapse, among others [156]. This might explain why the FERM and PDZ-containing SNXs only emerged in more evolved animals that present a complex nervous system.

In conclusion, the analysis proved robust (monophyletic groups corresponding for clades with specific domains) and showed a differential complexity (number and variability of SNXs) that was expected across different model organisms but more evident in animals. The use of a single representative genome allowed to better identify orthologs, homologs, and isoforms, according to their physical position in the genome.

## Sorting Nexins and the Nervous System

**Fig. 4:**
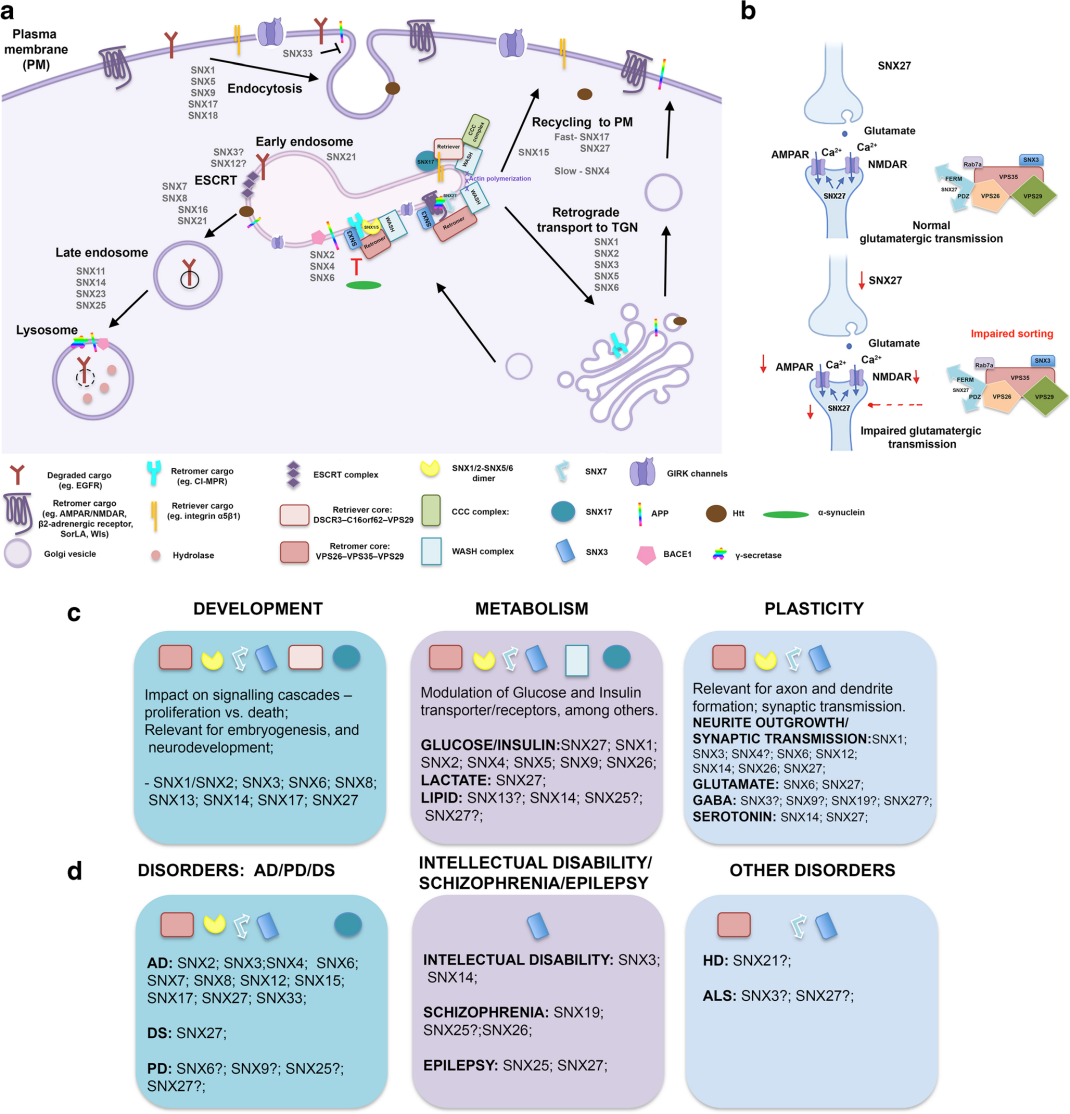
Schematic representation of SNXs interplay with distinct endocytic machineries/processes and their known/predicted roles on physiological and pathological processes. **a** Overview of proposed SNXs role on cargo sorting from the PM to distinct destinations: degradation; recycling and retrieval; from data including non-neuronal and neuronal cells. Representative SNXs and complexes of interest are shown in distinct trafficking routes of cargoes. Representative cargoes of relevance for neuronal function, and disease-associated, are also depicted, such as APP, BACE1, γ-secretase, α-synuclein, AMPA/NMDA receptors/Wls, GIRK channel, TrkB, among others. Of notice, the significance of SNX-BAR, SNX3, SNX17, SNX27 for the depicted processes, together with retromer, retriever and WASH involvement. The possible role of SNX3/SNX12 in orchestrating cargo sorting to the degradative pathway, in addition to SNX3 role in retrograde trafficking is also highlighted. **b** Example of SNX dysregulation impact on synaptic transmission. A synapse is represented together with the impact of SNX27 down-modulation on the reduction of AMPAR and NMDAR levels at the synapse, impacting on neuronal transmission. **c** Schematic representation of distinct SNXs and sorting complexes validated, or predicted, involvement in physiological processes relevant for nervous system development and function. **c** Summary of known/predicted associations between SNXs and sorting complexes and neurological disorders, spanning from genetic, developmental to neurodegenerative conditions. It's noteworthy the involvement of a wide array of SNXs with AD condition, highlighting novel molecular pathways for treatment. PXA-RGS-SNXs also seem pivotal for neuronal excitability modulation, which requires further validation. AMPAR, amino-3-hydroxy-5-methyl-4-isoxazolepropionic acid receptor; APP, amyloid precursor protein; BACE1, Beta-secretase 1; CI-MPR, cation-independent mannose 6-phosphate receptor; EGFR, epidermal growth factor receptor; ESCRT, endosomal sorting complexes required for transport; NMDAR, N-methyl-D- aspartate receptor; PM, plasma membrane; TGN, Trans golgi Network; Wls, Wntless

Since SNXs and their associated complexes sustain receptor trafficking (Fig. 4a, b), the contribution of these molecules to the regulation of the nervous system is obvious (Fig. 4c, d) (Table 1). We next described SNXs association with (neuro)developmental processes, brain metabolism, and synaptic plasticity, directly impacting on behavior.

### SNXs Role in (Neuro)Developmental Processes

In the context of development, different reports have associated SNXs with the modulation of receptors that control cell signaling events and cell proliferation. Accordingly, in pathological settings, SNXs mis-regulation leads to receptor signaling hyperactivation, or downmodulation, interfering with cell growth control and developmental programs, which have been observed in developmental disorders with marked neurological dysfunction [11, 16, 64, 130]. Specifically, SNXs involvement in developmental processes crucial for nervous system assembly and function has been reported by several groups and is depicted on Fig. 4 c. In particular, distinct SNX- mutants display embryonic lethality, or are non-viable at early stages of development, such as the double SNX1 and SNX2 KO [157, 158], SNX13 mutant [64], SNX14 mutant [24], and SNX27 mutant mouse models [142]. Other SNXs might also be of significance to development, but data is not yet available (Fig. 4c).

#### PX-Only Subfamily

Work by our group and by Harterink supports SNX3 involvement in development through its role in Wnt signaling regulation across phyla [24, 28]. SNX3, a retromer-associated SNX, is intrinsically linked to development in several studies performed in *D. melanogaster*, *C. elegans*, and in mammals [24, 28]. Harterink and co-workers elegantly demonstrated that SNX3 alone modulates Wnt signaling by sustaining Wntless (Wls) recycling through the retromer complex, as previously mentioned. Work by our lab also demonstrated that disrupting SNX3 alone lead to severe developmental defects, which were not present in the SNX1 mutant, or other SNX- mutants associated with the retromer complex (SNX1, SNX5, SNX27). Interestingly, we also showed that SNX3 mutation leads to severe neuronal wiring defects and to impaired neuro-dependent behaviors (such as locomotion), independently of retromer-sorting. This data suggests an important role for SNX3 in nervous system function, which will be further discussed on in this review in section (3.3). Furthermore, Mizutani et al showed that SNX3 regulates neurite outgrowth, and that it is strongly expressed in neural tissues throughout development and in adulthood [105, 159]. Recently, SNX3 was shown to mediate mammalian neural tube closure [111]. Mizutani also highlighted the role of the closely related PX-only, SNX12, in cerebral cortical development, which remains unexplored [114].

#### SNX-BAR Subfamily

Several BAR-containing and retromer-associated SNXs play roles in developmental processes (Fig. 4c). Particularly, SNX1 and SNX2 KO mouse models are viable and fertile, although double mutant embryos are arrested at mid-gestation suggesting a functional and redundant role for these retromer components in embryogenesis [158], similarly to Vps26 [160, 161], possibly due to alterations in nutrient receptor signaling. This phenotype was shown to be unrelated to SNX1- or SNX2-mediated trafficking of epidermal growth factor receptor (EGFR) [3, 43, 158] or CI-MPR [157, 161]. Co-localization of SNX1 SNX2 in the same endosomal structures is not clear [41, 43], and distinct reports highlight that their function is not interchangeable regarding cargo modulation [157, 158]. Purportedly, authors speculate that SNX1 would be engaged in cargo sequestering and SNX2 in retromer assembly and/or stability, being its mutation more detrimental for cell survival, or, that SNX1 regulates pathways that are not essential for embryonic development. This refutes the possible involvement of SNX1 on Wntless (Wls) recycling and Wnt signaling [162]. Furthermore, studies regarding the retromer-associated SNX5 KO in mice have highlighted that depletion of SNX5 causes no defect on developmental processes, since KO mice displayed normal life spans and fertility, although being smaller in size, compared to wild-type animals [163]. The data available regarding retromer-associated SNX6 depletion was gathered by analyzing a CNS-specific SNX6 KO mouse model, since a SNX6 full KO mouse has not been yet reported. Specific silencing of SNX6 with a Nestin-Cre expressing mouse model was shown to display mild neurodevelomental defects. The fact of it being a mild phenotype might arise from compensatory mechanisms involving other SNXs with overlapping functions, like SNX5 or SNX32 (that displays 85% similarity with SNX6). Interestingly, SNX6 is associated with BACE1 trafficking, which will be discussed in detail further in section 4.1. Recently, whole genome sequencing studies have also highlighted an association between SNX8, another BAR-containing SNX, with neurodevelopmental processes, which remains to be functionally verified [119] (Fig. 4c).

#### SNX-FERM Subfamily

SNX27 is another retromer-associated protein, but from the SNX-FERM subfamily, that plays a role in developmental processes. SNX27 levels are reduced both in DS patients and in DS mouse models [16]. In this congenital disorder, patients display multi-organ defects and severe developmental impairments and learning disabilities. The full mouse SNX27 KO was shown lethal, perishing around P14, supporting the relevance of SNX27 for development and survival, even that SNX27 is only expressed in postnatal days [16]. Of notice, Snx27 was identified as an alternative-splicing product of Mrt1 (methamphetamine responsive transcript 1) gene that was developmentally regulated and drug-inducible [141]. More recently, a study highlighted how SNX27 interaction with β-catenin, through its PDZ domain, can possibly shape Wnt signaling and, hence, embryonic developmental processes [164]. Furthermore, SNX27 associates with epilepsy [146] and patients with SNX27 variants display seizures, developmental delay, behavioral disturbance, and subcortical brain abnormalities [152]. Moreover, SNX27 could be indirectly shaping neurogenesis, and thus developmental processes, since it regulates the trafficking and subcellular localization (and thus availability) of the NMDAR, a known regulator of neurogenesis [165]. Other retromer-associated proteins, such as Vps26 and Vps35, have also established roles in stemness and neurogenesis.

SNX17, another SNX-FERM protein, is an adaptor for the retriever complex, and might also contribute for developmental processes, since this complex is evolutionarily conserved (as is the retromer) and plays a primordial role in the sorting of about 120 protein cargoes [89]. More so, SNX17 has been shown to modulate the trafficking of ApoER2, an important player of the signaling pathway that shapes cortical layering pattern of the mature neuronal cortex and is, therefore, crucial for development [166]. Simultaneously, SNX17 also regulates the Notch pathway, pivotal for cell fate determination. SNX17 acts as a cargo-specific adaptor for Jag1a, promoting its retromer-dependent recycling (possibly involving the retriever complex, which was unknown at the time) and Notch signaling and hence shaping cell fate determination and neurogenesis [132] (Fig. 4c). SNX17 can also possibly shape development through the regulation of the subcellular localization of the remaining identified cargoes [89].

#### SNX-PXA-RGS-PXC Subfamily

SNXs’ association with developmental processes does not end with its retromer-retriever-associated members. In fact, other SNXs are of relevance for development, such as those that belong to the PXA-RGS subfamily: SNX13 and SNX14. SNX13 was shown to play a crucial role in mouse development and in the regulation of overall endocytic sorting [64]. SNX13 full KO mice were embryonic lethal around mid-gestation, displaying several growth retardation and neural tube defects. Moreover, its endoderm cells present huge autophagic vacuoles and mis-localization of endocytic markers, suggesting overall endocytic trafficking impairments [64]. Interestingly, SNX13 expression is virtually absent in the brain [65]. On another note, although data concerning SNX14 full KO mice has not been published to date, SNX14 was highlighted as a gene of significance for neuronal development and function (Fig. 4c). Authors showed that SNX14 is ubiquitously expressed in the brain and that it’s silencing with shRNA approaches leads to impairments in synaptic transmission. Moreover, SNX14 was shown to associate with an intellectual disability syndrome, being involved in synaptic excitability, among others [11, 23]. Interestingly, synaptic dysfunction has been frequently implicated in neurodevelopmental disorders and can be associated with the disease implications of SNX14 mutations. Moreover, in the worm, a SNX14 ortholog deletion mutant is not viable [24], supporting a role for SNX14 in development and, of particular relevance, in nervous system function/maintenance.

In light of the above, it is reasonable to admit that a thorough understanding of SNXs functions will enable the discovery of new potential therapeutic targets for developmental disorders. This, however, requires a better understanding of SNXs’ involvement with developmental processes, namely of those SNXs whose function shape the nervous system, and of the contribution of their domain complexity to these processes. For that, scrutinizing the molecular interactions within this family (and their adaptors and effector proteins) with the signaling pathways that control cell proliferation and development is required.

### SNXs Described Role in Brain Metabolism

Our brain is extremely sensitive to alterations in energy metabolism. It requires a constant feed of nutrients, mostly glucose, to properly function. In fact, it comes with no surprise that abnormalities in energy metabolism are often observed in distinct pathologies that affect the CNS, such as developmental and neurodegenerative disorders, epilepsy, stroke, and migraine, among others. Glucose is indeed the main energy source for the brain, being approximately 25% of total glucose intake allocated for brain use. Other nutrients of relevance to the brain metabolism are the ketone bodies acetoacetate, lactate, and pyruvate. These carboxylic acids have been shown to promote synaptic activity in vitro, namely in isolated brain preparations [167]. Monocarboxylate transporters (MCTs) have been found in oligodendrocytes (MCT1s), and play a role in lactate export, while MCT4s, identified in astrocytes, enable high lactate transport rates, and MCT2s are found present in neurons [168]. Interestingly, authors hypothesize that lactate is an “opportunistic” glucose-sparing substrate when it is present in high concentrations within the brain [169], and it is possibly used when neurons are firing at high frequencies.

It comes with no surprise that by shaping the sub-cellular localization of different nutrient receptors distinct SNXs family members are implicated in the regulation of cellular metabolism, particularly from the SNX-BAR/SNX-SH3-BAR/SH3 and the SNX-FERM subfamilies, concerning glucose metabolism, and of the SNX-PXA-RGS-PXC subfamily regarding lipid metabolism.

#### SNX-BAR/SNX-SH3-BAR/SH3 Subfamilies

Several SNXs have been linked to the regulation of cellular metabolism, particularly of glucose metabolism, such as retromer components: SNX1, SNX2, and SNX5, and the SNX4, SNX9, and SNX26 [4, 140, 170, 171] (Fig. 4c). Interestingly, these SNXs display a BAR, a SH3 or a BAR-SH3 domain. SNX1, SNX2, SNX4, and SNX5, BAR containing SNXs were shown to coimmunoprecipitate with the insulin receptor, among others [4]. Particularly, SNX5 was demonstrated to regulate insulin receptor expression in human cells, and also its association with insulin-degrading enzyme (IDE) [171] and, via this mechanism, modulate insulin signaling and degradation [171]. SNXs were also shown to promote insulin secretion and function through the regulation of other hormones such as of glucagon-like peptide 1 (GLP-1), namely by SNX1 [172]. SNX9 on its turn was shown to potentiate the correct sorting of a glucose transporter (GLUT4) to and from the insulin responsive compartment, in a process dependent on its own phosphorylation state [170]. Authors have also demonstrated that SNX9 interacts with insulin receptors and with insulin-like growth factor 1, with an active role in the action of insulin in the cell [170] (Fig. 4c). Another SNX proposed to play a role in insulin-mediated glucose transport is SNX26 (also known as ARHGAP33 or TCGAP) [138]. A brain-enriched GTPase-activating protein, SNX26’s function has been poorly explored (Fig. 4c), although it was shown to be involved in insulin-mediated glucose transport and in GLUT4 translocation [138].

#### SNX-FERM Subfamily

In a comprehensive and elegant approach, Steinberg and colleagues identified over one hundred cell surface proteins that interact with a SNX of particular relevance for the nervous system — the SNX27 — from which, glucose and amino acid transporters, monocarboxylate transporters (MCT1), as well as many others, including signaling receptors were identified [58]. Glucose transporter 1 (GLUT1), or solute carrier family 2 facilitated glucose transporter member 1 (SLC2A1), is a protein that facilitates glucose transport across the plasma membrane of mammalian cells. In the brain, two isoforms occur: one is produced in the brain microvasculature, supporting glucose transportation across the blood brain barrier (BBB); and another in astrocytes, which supports glucose intake and consequent lactate production, providing alternative energy sources for neurons. Authors highlight that SNX27 promotes GLUT1 recycling to the cell surface through the retromer complex, potentiating glucose intake. When SNX27, or Vps35, expression is silenced the GLUT1 transporter is sorted for degradation in the lysosomes, and the amount of cellular glucose intake is significantly reduced [58] (Fig. 4c). Of notice, this was not observed when other SNXs that form the retromer complex were silenced, such as SNX1 and SNX5. Recently, Ding and co-workers also identified a role for the WASH complex in modulating GLUT2 trafficking in the islets of Langerhans and, in this manner, in sustaining whole-body glucose homeostasis [173]. Since the WASH complex has been intrinsically linked to the trafficking of both SNX17 and SNX27 dependent cargoes, via its interaction with the CCC retriever and retromer complexes, authors postulate that GLUT2 sorting occurs through a similar mechanism of endosomal retrieval, thus involving WASH, retromer, and SNX27 (Fig. 4c). SNX27 also modulates glucose consumption through its physical interaction with insulin receptor [174], and with the insulin-responsive glucose transporter GLUT4 [175, 176]. Therefore, when SNX27 expression is decreased, less GLUT4 receptor is available for activation. SNX27 also promotes insulin secretion and function through the regulation of the glucagon-like peptide 1 (GLP-1) [172]. Furthermore, SNX27 was shown to interact directly with the tumor suppressor PTEN [177], where, PTEN binding to SXN27 was shown to impair proper GLUT1 recycling to the cell surface, by blocking retromer assembly, and hence directly impacting on glucose intake by the cell. This is of relevance for tumor settings, as a healthy cell relies ion PTENS’ ability to modulate GLUT1 levels at the plasma membrane, modulating glucose intake levels and, hence, sustaining its tumor suppressor ability [177]. In fact, PTEN mutations have been widely annotated in a variety of tumors, namely in brain tumors such as gliomas [178]. SNX27 was also linked to the MCT1 transporter trafficking regulation, preventing its sorting to the degradative pathway [58]. Nevertheless, it remains to be elucidated if silencing of SNX27 expression has a direct impact on lactate consumption by the cells, and if it is of relevance to the nervous system. More recently, SNX27 was shown to modulate the trafficking of the alanine, serine, cysteine-preferring transporter 2 (ASCT2; SLC1A5), which is responsible for glutamine uptake, a major energy source for the cell and also of significance to the mammalian target of rapamycin (mTOR) activation [179]. Glutamine is an important amino acid for metabolism, found abundant in the CNS and in the cerebrospinal fluid (CSF) [180]. It can serve as substrate for the synthesis of excitatory and inhibitory neurotransmitters, and also sustain energy-producing processes, such as the tricarboxylic acid cycle (TCA), among others. By regulating glutamine intake by the cells, SNX27 can also modulate cellular metabolism from a different pathway. Interestingly, SNX27 is also a known regulator of metabolic processes important for T-cell activation and immuno synapse formation, through its interaction with diacylglycerol kinase ζ (DGKζ), a negative regulator of Diacylglycerol (DAG) [181]. Accordingly, SNX27 could also be shaping lipid metabolism within the nervous system by controlling DAG formation, which remains to be studied. In this line, a thorough understanding of SNX27 cell-specific role is required, as this major regulator of homeostasis is involved in the sorting of more than 100 cargoes, and seems to be associated with panoply of conditions that severely impact on health.

#### SNX-PXA-RGS-PXC Subfamily

SNX19, from the PXA-RGS subfamily, is also a SNX associated with insulin signaling; specifically, its reduction decreases insulin secretion [182]. In parallel with the above-mentioned SNXs role on insulin signaling and function, spanning distinct subfamilies, and taking into consideration insulin’s association with brain homeostasis impacting on learning and memory, longevity, and age-related disorders, among others, it is tempting to speculate that SNXs association with neurological conditions might also arise from insulin-dependent metabolic dysregulation.

On another note, lipid metabolism within the brain is also of relevance for its function. Lipids (and intermediates) are major constituents of the brain, and compose approximately 50% of the brain dry weight. They are essential structural components of cell membranes, having an important role in neuronal structure and plasticity. Approximately 20% of total brain energy requirement is fulfilled by fatty acid oxidation [183, 184], with fatty acid metabolism of relevance for neurodevelopment, neurotransmission, and repair processes [185]. To date, only the PXA-RGS subfamily of SNXs has been directly linked with fatty acids metabolism. Studies in the fly identified the gene *snazarus* (*snz*), homolog to mammalian SNX13, SNX14, and SNX25, as highly expressed in fat tissues and involved in longevity [186] (Fig. 4c). More recently, *snz* was shown to regulate lipid droplet organization and to promote inter-organelle crosstalk (curiously, *snz* mammalian counterparts’ expression increases with adipogenesis and obesity). When *snz* expression is altered, lipid droplet (LD) organization is perturbed, as is the tryacylglyceride production, leading to starvation and, consequently, decreasing the fly lifespan [187]. Interestingly, studies highlight that SNX14 is a marker for ER-LD contacts and it is pivotal for the fatty acid stimulated LD growth in mammals, like its yeast homolog (Mdm1), implying a conserved function in fatty acid metabolism across species. Moreover, SNX14 has also been linked to neutral lipid metabolism, particularly to that of sterols, such as cholesterol [131] (Fig. 4c). Evidencing for SNX14’s role in lipid metabolism and homeostasis, when it is mutated cholesterol levels increase in late endosomal/lysosomal structures, but whether this is of relevance for brain function remains to be further verified. It also remains to be elucidated whether other PXA-RGS SNXs are of relevance for lipid metabolism. Nevertheless, attending on the significance of lipid homeostasis for neuronal survival, and on the growing association between neuropatholgies such as AD, autism spectrum disorders, DS, PD among others, and abnormal lipid metabolism [188, 189] and SNXs function, it is not surprising that other SNXs are pivotal for lipid metabolism homeostasis regulation, particularly those that underlie PIP dysregulation.

In summary, it is evident that there are numerous ways by which SNXs can influence cellular metabolism, particularly of nervous cells and of its main fuel, glucose. As above-mentioned, distinct SNXs members are postulated or already shown to be involved in distinct diseases, such as cancer, developmental and neurodegenerative disorders. The impact of SNXs mis-regulation in brain metabolism and its consequences for brain function are thus important fields of study that can contribute to the elucidation of the molecular mechanisms underlying different brain pathologies. This subject will continue to be discussed throughout this review.

### SNXs and Synaptic Plasticity

The brain is not a static organ, in fact it can be considered “a lifelong work in progress.” The brain changes by pruning or strengthening connections (synapses), depending if unnecessary or important to retain, thus building specialized brain circuits. This concept is regarded as plasticity, or more specifically, synaptic plasticity, where chemical synapses can modulate their strengths according to their perception of environmental stimuli, or while recovering from noxious insults. This phenomenon is the major cellular mechanism that underlies learning and memory. Due to the increased protein concentration levels required to sustain potentiation processes, and so to retain information, structural changes are in order and demand synaptic enlargement.

We will now discuss how distinct SNXs are involved in synaptic modulation, by interfering both with dendritic remodeling and neurotransmission, crucial events for the maintenance of plasticity, and also on how they can impact on the functionality of distinct brain regions. Simultaneously, we will address the significance of SNXs for these processes.

#### Dendritic remodeling and synaptic transmission

Synaptic inputs are perceived by neuronal projections called dendrites, which are critical for information transmission and signal propagation. Multiple synapses emerging from different axons can convey information to a dendritic tree, which will combine signals. In this manner, dendritic spines undergo activity-dependent structural remodeling that is pivotal for learning processes and for memory. Trafficking machinery has gained a role of relevance for these processes since the availability of recycling endosomes at the base of the dendritic spines enables the fast turnover and recycling of receptors sustaining the establishment of long-term potentiation (LTP) and long-term depression (LTD), as determined by the environmental context [190]. Multiple players are known to promote dendritic pruning involving branch retraction or extension.

##### PX-Only Subfamily

The retromer itself has been shown to localize to neuronal cell bodies, axons, and dendrites, being pivotal for synaptic morphology, transmission, and even synaptic vesicle content [191]. Regarding the retromer-associated SNXs, work from Mizutani and colleagues highlighted a strong SNX3 signal in cerebellar Purkinje cells at the postnatal stage, when the outgrowth of their dendritic branches initializes [159]. The same expression pattern was identified in the hippocampus at the embryonic stage, when axonal growth is high. Thus, authors hypothesize SNX3 may induce axon/dendrite formation [159] (Fig. 4c). Furthermore, SNX3 was shown to influence synaptic connectivity by regulating neurite formation. Transfection of N1E-115 and neuroblastoma cells with SNX3 shRNA decreased lithium-induced outgrowth of neurites, whereas its upregulation facilitates outgrowth [105]. SNX3 close homolog SNX12 was also associated with neurite outgrowth regulation during cerebral cortical development. Studies with the same neuroblastoma cells, and in rat primary cortical neurons, highlight that SNX12 expression increases as neurite outgrowth occurs and that SNX12 down-modulation attenuated its growth [114] (Fig. 4c).

##### SNX-BAR Subfamily

Furthermore, in neuroblastoma cells (N2a), expression of retromer-associated SNX1 also induced neurite outgrowth, which was potentiated by its co-expression with EFA6A. The authors propose a novel mechanism where EFA6 regulates Arf6-mediated neurite formation through SNX1 [192]. SNX6 also plays a role in dendritic remodeling. A mouse model homozygous for a neuronal-specific conditional allele displays decreased dendritic spine density in the CA1 neurons in the hippocampus and impaired spatial learning and memory [117]. SNX6 was demonstrated to interact with Homer1b/c, a postsynaptic scaffold protein crucial for synaptic distribution of postsynaptic density (PSD) proteins and for the structural integrity of synaptic spines (Fig. 4c). SNX6 ablation affected Homer1b/c distribution on dendrites and impacted on glutamate receptors, AMPAR, localization and, thus, on synaptic transmission and plasticity [117]. Recently, SNX4 (an ubiquitously expressed brain protein) was also shown to strongly co-localize with synaptic markers and found present both at the pre- and post- synaptic terminals. Authors postulate that SNX4 is aiding the endolysosomal system in the regulation of both the insertion of metabotropic receptors, like G-protein coupled receptors (GPCRs), important regulators of synaptic communication, as well as of the insertion of neurotransmiter receptors at the post-synaptic site, shaping synaptic plasticity [193].

##### SH3 Subfamily

SNX26 (also known as ARHGAP33), a poorly studied and brain enriched SNX, is also involved in dendritic spine formation in mature neurons [139, 140]. SNX26, a GTPase-activating protein, interacts with PSD-95 and plays a role in activity-dependent dendritic spine formation. Furthermore, SNX26 was also shown to regulate TrkB trafficking, a high-affinity receptor for brain-derived neurothrophic factor [140], shaping dendritic development (Fig. 4c). Interestingly, SNX26 KO mice display impaired neurite outgrowth among other abnormalities [140].

##### SNX-FERM Subfamily

SNX27 is by far the most brain- and pathology-related SNX, possibly due to its PDZ domain, a protein-protein interaction domain often found in proteins of relevance for excitatory synapses (and, therefore, has also been intrinsically associated with synaptic transmission). In the brain, SNX27 is found primarily in dendrites and spines [194] and regulates synaptic plasticity (Fig. 4c). SNX27 modulates glutamate transmission by targeting NMDAR and AMPAR to the cell surface [16, 194]. Indeed, SNX27 dysfunction has been extensively shown to underlie excitatory synaptic defects associated with DS and epilepsy [16, 146]. Furthermore, SNX27 modulates surface levels of G protein-gated rectifying potassium (GIRK) channels, thus orchestrating neuronal excitability [195]. More recently, trafficking of neuroligins (NLGs), postsynaptic *trans*-synaptic scaffold proteins important for synaptic stability, was also shown to be dependent on SNX27 and retromer components [196]. Upon SNX27 neuronal depletion, NLGs 1-2-3 levels are reduced, as their lysosomal degradation is favored, impacting on inhibitory synapse number and, consequently, shaping synaptic transmission. While SNX27 contribution to excitatory synapses function has been extensively explored, SNX27 characterization, as a potential regulator of inhibitory synapses, remains poorly characterized. Still, SNX27 pivotal role in NLGs trafficking unveils a putative role for SNX27 in pathologies that display aberrant inhibitory synaptic transmission, like mood disorders, epilepsy, and autism, among others. Recently, SORLA, a SNX27 binding partner tightly associated with AD, was also shown to modulate neurite outgrowth [197]. SORLA overexpression in cultured neurons potentiated neurite development and accelerated neurite regeneration, possibly through the activation of EGFR/ERK/Fos signaling cascade by soluble SORLA [197]. SNX27 binds to the cytosolic tail of SORLA promoting its translocation to the cell surface [147] and, in this manner, it can purportedly modulate the amount of soluble SORLA available and, hence, its neuroprotective role. Furthermore, SNX27 also interacts and regulates GPR17 trafficking and, thus, oligodendrocyte differentiation [148]. Attending on the relevance of neuronal-glia communications for synaptic function, and on the recently ascribed roles for oligodendrocites in the modulation of neurotransmitter release at presynaptic terminals through the secretion of brain derived neurotrophic factor (BDNF) [198], SNX27 might also be shaping synaptic transmission through this pathway.

Other SNXs, which are not hitherto retromer associated, are also known to shape neurite morphology and synaptic transmission. For instance, SNX17 was associated with dendrite tree development and with reelin signaling [199]. Data obtained with transgenic cultured neurons highlight a role for SNX17 in linking endocytic trafficking with receptor signaling, being its expression important for the modulation of brain development and function, namely for synaptic plasticity and learning [199].

##### SNX-PXA-RGS-PXC Subfamily

SNX14 is a well-known regulator of neuronal excitability, promoting synaptic transmission [23] (Fig. 4c). SNX14 downmodulation was shown to reduce intrinsic excitability and to markedly impair both excitatory and inhibitory synaptic transmission. Moreover, mutations in SNX14 are of relevance for cognitive performance [11]. SNX25 also recently emerged as a novel player for BDNF-TrkB signaling in the CNS, being possible of relevance for synaptic transmission [68], which needs to be further explored.

#### Glutamatergic transmission

Undoubtedly, glutamate is the most abundant neurotransmitter in the brain and is a pivotal excitatory player of synaptic plasticity (hence, central for learning and memory processes). Glutamate is also important for the synthesis of GABA and, thus, indirectly for inhibitory processes. Imbalances in glutamatergic transmission trigger seizures and excitotoxicity, common mechanistic features of neurodegenerative conditions such as AD and inherent to aging. Glutamate transmission relies on a subset of glutamate receptors that can be categorized as metabotropic and ionotropic [200]. Trafficking of glutamate receptors has been extensively studied, attending on the significance of LTP establishment for memory and learning processes, which are essential for survival, namely throughout evolution.

##### SNX-BAR Subfamily

The retromer and SNXs have been tightly associated with these events. Particularly, in immature hippocampal neurons, where the constitutive delivery of AMPA receptors has been shown to rely on retromer components such as Vps35 and SNX6 [117, 201], and in mature neurons, where AMPA trafficking dependence on retromer and SNXs seems restricted to the triggered pathway [202]. Specifically, SNX6 depletion was shown to induce a reduction of Homer1b/c in distal dendrites, and to decrease the surface levels of AMPAR, impairing in this way synaptic transmission [117] (Fig. 4c). This phenomenon is retromer independent.

##### SNX-FERM Subfamily

SNX27 is reported to interact both in vitro and in vivo with AMPAR and NMDAR, being pivotal for the establishment of LTP [16, 194]. The authors highlight a major role for SNX27 in the trafficking of AMPARs, coupling plasticity stimuli to the delivery of postsynaptic cargo (Fig. 4c). Briefly, through a Ca^2+^/CaM-dependent mechanism, K-ras GTPase is recruited to endosomes where it interacts with SNX27 promoting synaptic delivery of homomeric GluA1 receptors [194]. As such, SNX27 shapes the distribution of GluR1, GluR2, and GluA1, but not GluA2 (at least directly) [16, 194].

#### GABAergic Transmission

The *γ*-aminobutyric acid (GABA) is a crucial regulator of neuronal networks, being the most abundant and well-studied inhibitory neurotransmitter in the mammalian nervous system. GABAergic transmission is crucial for neurodevelopmental processes, and its dysregulation is strongly linked with developmental diseases [203]. In addition, GABAergic transmission gained relevance in the context of neurodegenerative processes. Multiple models of neurodegenerative disorders, like AD and PD, among others, display impairment of GABAergic interneurons and their inherent excitatory (inhibitory neurotransmitter balance [204]. GABA transmission relies on GABA action on GABA receptors [205] whose distribution is susceptible for regulation by protein trafficking effectors such as SNXs.

##### PX-only subfamily

SNX3 was recently shown to shape GABAergic neuronal architecture and wiring [24]. Using *C. elegans* as a model, we validated SNX3 role in neurodevelopmental processes as well as in neuro-dependent behaviors, such as locomotion, and highlighted how *snx-3* mutation affected the neuronal structure of GABAergic neurons and their ramifications (Fig. 4c).

##### SNX-FERM Subfamily

Interestingly, SNX27 has also been associated with GABA transmission through its role on GIRK channels trafficking regulation [195] (Fig. 4c). Similarly to other neurotransmitters such as serotonin and adenosine, GABA is known to drive G protein-dependent activation of GIRK channels, potentiating inhibition of neuronal activity. GIRK channels activation reduces membrane excitability, resulting in inhibition of neuronal firing rate. SNX27, through its PDZ domain, regulates GIRK channels surface levels by potentiating their recycling from early endosomes to the cell surface, requiring a functional Ras domain to do so [195]. Interestingly, when SNX27 levels are decreased, GABA receptor-GIRK currents are reduced, potentiating drug-sensitization effects. In vitro, SNX27 overexpression also indicates that GIRK currents are lower, possibly through channelling GIRK for lysosomal degradation [206]. This establishes a link between GIRK channels trafficking regulation, slow inhibition and the functionality of reward circuitries, which should be further explored in new therapeutic targets for reward-associated conditions such as drug addiction.

##### SNX-PXA-RGS-PXC Subfamily

SNX19 has also been associated with conditions that display GABAergic dysfunction, such as schizophrenia (which will be later discussed in section 4.2). Upon SNXs depletion, there might be shared molecular mechanisms susceptible to disruption, found in schizophrenia, as well as in other GABA-dependent conditions, such as developmental disorders.

#### Serotonergic signaling

Serotonin (5-hydroxytryptamine, 5-HT) is a neurotransmitter often associated with the pathophysiology of various neuropsychiatric disorders. Serotonin exerts its function both at the central and peripheral levels, and is important for several processes such as thermoregulation, food intake, sleep/wake cycle, nociception, locomotion, mood and social cognition, neurogenesis, memory, gastrointestinal function, and cardiovascular regulation, among others. Serotonin pleiotropic role is mediated by a family of serotonin receptors which comprise not less than 14 GPCRs and a combination of ligand-gated cation channel heteropentameric receptors [207, 208].

##### SNX-FERM Subfamily

SNX27 has been identified as of relevance for 5-HT_4_R sorting, a receptor involved in learning and memory processes [143]. Authors made evident that both the constitutive and methamphetamine induced isoforms of SNX27, *a* and *b*, respectively, interact with this serotonin receptor promoting its sorting into early endosomes [143].

##### SNX-PXA-RGS-PXC Subfamily

Within the SNX family, the most obvious candidates as regulators of serotonin signaling are the members of the PXA-RGS subfamily: SNX13, SNX14, SNX19, and SNX25 (all known regulators of G protein signaling). In fact, SNX14, a brain-enriched SNX, has been categorized as a dual endogenous negative regulator of neuronal 5-HT_6_ receptor signaling, a receptor important for cognition and anxiety [65] (Fig. 4c), by inhibiting signaling and trafficking of 5-HT_6_R. On one hand, SNX14 interaction with the receptor promotes its degradation, and on the other hand its RGS domain associates and sequesters Gαs, inhibiting downstream cAMP production. Phosphorylation of SNX14 by protein kinase A (PKA) was shown to inhibit SNX14 association to Gαs, rendering SNX14 available for binding to 5-HT_6_R and, thus, facilitating its internalization and degradation [65]. SNX13 is closely related to SNX14, and although it shares Gαs regulation ability, being actively associated with cAMP signaling regulation, it is virtually unexpressed in the brain. Also, no clear associations have been established with serotonin receptor signaling. Lastly, SNX19 is deprived of a RGS domain, and SNX25 has been linked to dopamine receptor trafficking and TGF-β receptor degradation, with no evident association with serotonin receptor noted for either of these.

Overall, it is evident that SNXs from distinct subfamilies contribute to nervous system homeostasis, sustaining processes of relevance for development, metabolism, and for neuronal connectivity/circuitry. Attending on SNXs family domain complexity and to the higher number of SNXs occurring only in animals, it is tempting to speculate that many more associations will arise between SNXs function and nervous system regulation as distinct orthologs are being characterized. Additionally, SNXs contribution to the nervous system in a cell- or brain region-specific manner has not been fully explored, such as SNXs role within glial cells for instance. Exciting discoveries are thus surely ahead. Undoubtedly, the assessment of SNXs impact on convergent molecular pathways that shape brain function and circuitry organization will be important to understand the circuit mechanisms that drive behavior and their regulation, which is particularly important to identify the origins of illness, namely in the face of adverse settings.

## Sorting Nexins and Nervous System Pathology

Distinct studies have highlighted that both downregulation and upregulation of several SNX family members are observed in conditions that affect (neuronal) health, with some even establishing causative links (Fig. 4d). We will now discuss the relevance of SNXs imbalance for brain health, hoping to pinpoint targets for new therapeutic avenues.

### SNXs Association with Alzheimer’s Disease/Parkinson’s Disease/Down’s Syndrome

Distinct members of the SNXs family have been associated, either directly or indirectly, with a wide array of pathologies, including those of the/that affect the, nervous system and brain function (such as AD, PD, and DS) [1, 2, 11, 14, 16, 23, 114, 209].

AD is the most common neurodegenerative disorder, being characterized by the accumulation of Aβ plaques, and of intracellular neurofibrillary tangles (NTFs), particularly in the hippocampus and in the cortex. NTFs are composed of phosphorylated filaments of the microtubule-associated protein TAU, and Aβ plaques are generated in the amyloidogenic pathway, by the sequential proteolytic cleavage of the β-amyloid precursor protein (APP) by the β- and γ-secretases, resulting ultimately in the Aβ peptide accumulation which is toxic. APP can also be cleaved in the non-amyloidogenic pathway by the α-secretase, forming a large soluble ectodomain of APP, which is non-toxic for the cell and exhibits neurothrophic and neuroprotective functions [210]. In the amyloidogenic pathway, β-secretase activity is mediated by β-site APP-cleaving enzyme 1 (BACE1), whereas γ-secretase activity is performed by a multi-subunit tansmembrane protein complex that includes, among others, the presenilin components PS1 and PS2 [211]. Growing evidence supports that the subcellular localization and the intracellular trafficking of APP, and of the amyloidogenic proteases, is crucial for Aβ accumulation, which confers a pivotal role for the trafficking machinery that regulates these cargoes, especially in pathological settings.

#### PX-only Subfamily

SNX3, a PX-only subfamily member, was recently linked with AD pathology, with studies involving its overexpression indicating that APP steady-state levels at the cell surface are favored. Moreover, SNPs in SNX3 have also been identified in AD patients [15]. In pathological settings, abnormal SNX3 expression can thus facilitate APP internalization and thus Aβ production. In addition to the relevance of APP mis-sorting for AD, the intracellular trafficking of the amyloidogenic proteases also emerged as critical to the AD pathology. β-Secretase-mediated (BACE1) APP cleavage has been demonstrated to constitute the limiting step in Aβ production, and BACE1 trafficking has been extensively linked to AD condition [212, 213]. A growing number of SNXs have been associated with APP intracellular trafficking regulation and with the modulation of BACE1 and γ-secretase activities. SNX12, a SNX highly expressed in the brain [114] closely related to SNX3, also modulates Aβ production by promoting cell surface steady-state levels of BACE1 [12, 114]. In this manner, SNX12 is reduced, as in the brains of sporadic AD patients [12, 209], and thus BACE1 internalization is favored, promoting Aβ accumulation. SNX12 is also regarded as an aging biomarker that associates with AD [127].

#### SNX-BAR Subfamily

SNX4 and SNX6 have also been shown to interact with the β-secretase BACE1 in the early endosome and to promote BACE1 trafficking, thus modulating Aβ levels (Fig. 4d). SNX6 knockdown promotes retrograde transport of BACE1 from the cell surface enhancing BACE1-derived Aβ production [115], and suggest that this retromer component negatively modulates BACE1-mediated APP cleavage [115]. SNX6 expression is also decreased in the brains of AD patients [115]. SNX4, on another note, was shown to prevent BACE1 degradation (thereby aiding in BACE1-endosomal stabilization and promoting Aβ accumulation [112]). Other BAR-containing SNXs were also associated with APP misprocessing. Particularly, SNX7 was linked with the AD condition, with authors highlighting that SNX7 decreases Aβ production by modulating APP cell surface levels [52] (Fig. 4D), as was SNX8, where SNPs have been linked with the risk of development of Late onset AD [214], being SNX8 also identified as an Aβ toxicity enhancer [118, 214] (Fig. 4d). SNX8 expression was shown to be decreased in human and mouse AD brain [53]. In a neuroprotective role, it is suggest that SNX8 enhances non-amyloidogenic APP trafficking.

#### SNX-SH3-BAR Subfamily

On a similar note, SNX33, as SNX9, where shown to modulate cell surface levels of APP by inhibiting APP endocytosis through its interaction with dynamin, aiding in the retention of APP at the plasma membrane and potentiating APP processing by the α-secretase in the non-amyloidogenic pathway [14] (Fig. 4d). Interestingly, it is noteworthy that several of the APP and BACE1-interacting SNXs belong to the PX-BAR or SNX-SH3-BAR subfamilies such as: SNX4, SNX6, SNX7, SNX8, SNX9, and SNX33 (the subgroup). In addition, SNX15 (a poorly characterized SNX belonging to the “Other SNXs subfamily”) was also shown to promote APP recycling back to the cell surface, diminishing the chances of Aβ production [69] (Fig. 4d). Although SNX15 is deprived of a known BAR domain, it has been reported to form homodimers with the BAR-containing SNX1, SNX2, and SNX4. SNX1 and SNX2 are also reported to display SNPs or reduced expression in the brains of AD patients [15], with SNX2 expression increased in aged healthy mice [101]. Undoubtedly, the BAR domain plays an important role in the AD condition, possibly through associations with the retromer (or other sorting) complexes, which could potentially be explored in therapeutics.

#### SNX-FERM Subfamily

The first SNX to be linked to the AD pathology was a FERM-containing SNX - SNX17 [13], being shown to promote steady-state cellular surface APP levels. When SNX17 was silenced, or its levels down-modulated, APP surface levels were diminished and Aβ production increased [13] (Fig. 4d). Moreover, SNX17 indirectly shapes APP processing and Aβ clearance, since it regulates the low-density lipoprotein receptor 1 (LRP1) trafficking and promotes its cell surface distribution [59]. LRP1 is a receptor known to bind APP and to modulate its amyloidogenic processing [215]. Finally, the brain-enriched SNX27 has also been extensively linked with the AD pathology by interfering with γ-secretase activity and thus with Aβ levels [145] (Fig. 4d). Concretely, SNX27 interacts with PS1/ γ-secretase, inhibiting its proteolytic activity and, thereby, Aβ production [145]. Moreover, although no direct interaction has been reported between SNX27 and APP, proteomic data indicates that APP levels are markedly reduced when SNX27 is depleted [58]. Emerging data suggests that SNX27 interacts with SORLA forming a ternary complex with APP [147], aiding SORLA in the retrieval of endosomal APP through the retromer complex, to the cell surface, promoting the non-amyloidogenic pathway [147]. Interestingly, lower levels of SNX27 have also been reported in AD patients, but its downmodulation seemed not to exacerbate amyloidogenesis in an AD mouse model [150]. On another note, SNX27 has also been tightly linked with DS [16]. In fact, authors showed that when SNX27 expression is reduced, animals display synaptic dysfunction and cognitive impairments [16]. Briefly, in a DS mouse model (Ts65Dn) occurs an overexpression of the chromosome 21-encoded micro RNA miR-155 that decreases C/EBPβ, a transcription factor responsible for SNX27 transcription, thus attenuating SNX27 levels [16] and glutamate receptor trafficking, leading to synaptic deficits. Directed SNX27 expression in the hippocampus of the DS animals strikingly restores cognitive performance [16]. With DS patients develop AD at older ages [216], possibly, this SNX27 mis-regulation in DS settings can contribute to, or potentiate, the development of AD during aging. PD is the second most common neurodegenerative disorder, affecting 1–3% of the worldwide population over 65 years old. There are two neuropathological hallmarks in this disease: the progressive and selective dopaminergic neuronal loss in the mid-brain region (substantia nigra par compacta - SNc) [217], and accumulation of intracellular inclusions of aggregates — Lewy bodies — mainly formed by α-synuclein [110, 218–220]. PD is a multifactorial disorder, with the underlying molecular mechanisms involved in its pathogenesis implicating distinct pathways, namely those that mediate α-synuclein proteostasis, and also mitochondrial function, oxidative stress, axonal transport, and neuroinflammantion, among others [221]. Recently, the retromer complex has emerged as of relevance to PD [21, 22, 222–224]. Particularly, both in familial and sporadic cases, a missense mutation in the retromer component Vps35 (Vps D620N) has been markedly linked to the late-onset form of PD [225, 226]. Authors showed that this mutation did not however impair the assembly of the retromer recognition complex, and hypothesize that the link between Vps35 and PD most probably relies on the direct interaction between Vps35 and the PD-associated gene Parkin [227]. Indirectly, this mutation alters retromer association with the WASH complex, and hence interferes with general cargo recycling, such as that of the dopamine receptor [228], and also with autophagosome formation [229], impacting on PD development. Thus, the retromer complex not only shapes protein degradation and recycling but also interferes with lysosomal health, association with other sorting complexes and with autophagy. Retromer also regulates Wntless trafficking, as previously referred throughout this review, a receptor associated with dopaminergic neuronal health and PD [230]. Nevertheless, and in the context of this review, although the Vps retromer components have been tightly linked to PD, studies highlighting a direct association between SNXs and PD, including those involved in retromer function, are scarce or inexistent, suggesting that SNXs are not relevant to this condition, until date. Still, some aspects are worth being highlighted, as follows.

#### PX-only Subfamily

Recent work by Patel and co-authors highlighted that in yeast and worm PD models α-synuclein blocks SNX3 association with endosomes impairing the retromer-dependent recycling of iron [110] (Fig. 4d). Authors showed that α-synuclein disrupts iron homeostasis in dopaminergic neurons by inhibiting SNX3-retromer function [110]. This work highlights, for the first time, the association of a SNX with the PD condition, opening new therapeutic avenues. Interestingly, work from our group has also shown that a SNX3 worm deletion mutant displays severe neuronal wiring defects and motility impairments [24]. This is however not surprising if taking into consideration the reported role of SNX3 in retromer recruitment and stabilization on endosomal membranes, through a direct interaction with Vps26 and Vps35 [231], and the roles of the cargo recognition Vps trimer in the PD condition. It is also noteworthy that the brain-enriched SNX12, which resembles SNX3 structure, also associates with Vps26 and that its silencing was shown to affect retromer endosomal localization [31]. This suggests that much is still unknown about SNXs and PD, and that besides Vps35 other genes can also become targets of interest in an effort to further modulate this pathology (Fig. 4d).

#### SNX-BAR Subfamily

GWAS studies have also highlighted a link between mutations in several genes and PD susceptibility, such as the previously mentioned Vps35 and also Parkin, Pink1 and LRRK2, among others [232]. Such establishes a possible link between the proteins that regulate their trafficking and PD. In fact, the BAR-containing and retromer component SNX6 was shown to directly interact with Rab32, which indirectly regulates LRRK2 sub-cellular localization [233, 234] (Fig. 4d). Both Rab32 and LRRK2 mutations have been associated with inherited and sporadic forms of PD [233, 235], although it remains unclear how the retromer and LRKK2 are linked in the context of PD. Other gene of interest for PD is Parkin, an ubiquitinase ligase important for mitochondrial function [236] that has also been linked to SNX9 turnover [237]. SNX9 (SNX-SH3-BAR subfamily) is important for vesicle release and was shown to regulate synaptic vesicle endocytosis and retrieval [120–122] (Fig. 4d). Whether this is of relevance to the context of PD remains to be elucidated.

#### SNX-FERM Subfamily

Additionally, SNX27, another retromer-associated SNX, can also potentially play a role in PD (Fig. 4d). Rifkin and co-workers recently demonstrated that SNX27 regulates GIRK currents, both in the ventral tegmental area (VTA) and SNc dopamineirgic neurons, and hence the excitability of dopaminergic midbrain neurons [206]. Interestingly, alterations in the excitability of midbrain dopaminergic neurons are major components of the sub-cellular alterations that underlie, among other conditions, neurological disorders such as PD and epilepsy [206].

#### SNX-PXA-RGS-PXC Subfamily

Recently, SNX25 was shown to be of relevance for TrkB receptor degradation [68] and for BDNF-TrkB signaling in the CNS, opening possibilities for SNX25 association with conditions that displayed impaired BDNF-TrkB signaling like PD.

### SNXs Role in Intellectual Disability, Ataxia, Schizophrenia and Epilepsy

Attending on all the above-mentioned roles of SNXs in the regulation of molecular processes involved in nervous system development, neuronal excitability, synaptic-development, -strength and -transmission, it is not surprising that SNX dysregulation has been associated with conditions that display cortical abnormalities, impaired circuitry, and disruptive physical and psychological features (Table 1). Distinct SNX family members have been linked to conditions such as schizophrenia and epilepsy, with major incidence in the SNX-PXA-RGS-PXC subfamily. Still, other associations were also established as further highlighted.

#### PX-only Subfamily

SNX3 gene was associated to microcephaly, microphthalmia, ectrodactyly, and prognathism (MMEP) and mental retardation [104]. However, analysis of a set of additional patients did not support this association [238]. Authors however highlight that mutations/deletions at SNX3 locus could be underlying abnormal cortical development in some patients, as reported by Vervoort, impacting on cognitive performance.

#### SH3 Subfamily

The poorly characterized SNX26, or ARHGAP33 (alias), is a well-established regulator of TrkB (a high-affinity receptor for brain-derived neurotrophic factor) and intracellular trafficking [140]. SNX26 mutation was shown to lead to severe spine maldevelopment and neuropsychiatric disorder-related behavioral abnormalities (Fig. 4d). Curiously, SNX26 expression is decreased in patients with schizophrenia [139], highlighting once again the multitude by which SNXs dysregulation can interfere with cellular physiology.

#### SNX-FERM Subfamily

The brain-enriched SNX27 has also been linked to epilepsy [146] (Fig. 4d). Authors observed that distinct variants of SNX27, possibly non-functional, occur in patients that display seizures, developmental delays, subcortical brain abnormalities, epilepsy, dysmorphic features, and impaired behavioral responses, among others [146]. By correlating in vivo and in vitro information, it is postulated that SNX27 might be exerting its role through distinct neuroactive mediators, such as the SNX27-dependent neuroreceptors: AMPA, NMDA (DS, cognitive disability), 5-HT (mood disorders - anxiety; social behavior), mGluR5; the ATPase copper transporting alpha (Menkes disease), the glucose transporter 1 (GLUT1 - infantile epilepsy), the disintegrin and metalloproteinase domain-containing protein 22 (ADAM22-invovled in encephalopathy, cortical atrophy and epilepsy); neuroligin 2 (autism, intellectual disability, anxiety); as well as with the recently described neuronal enriched cargoes that highlight SNX27 close association with membrane excitability and synaptic plasticity regulation [152, 193] (Fig. 4d).

#### SNX-PXA-RGS-PXC Subfamily

Several members of the PXA-RGS subfamily have been identified as pivotal for neuronal development and neuronal excitability regulation, among others, being of relevance for normal brain development/function (with impact on “intellectual fitness”). Specifically, mutations in SNX14 were shown to underlie a distinctive autosomal-recessive cerebellar ataxia and intellectual disability syndrome [11] (Fig. 4d). Co-expression network analysis (WGCNA) enabled the identification of the subset of genes co-regulated with SNX14, unveiling an association between SNX14 and genes involved in vesicle-mediated transport between the ER-Golgi as well as with genes associated with cellular protein metabolism [11]. Further studies identified biallelic SNX14 mutations in 12 families with cerebellar atrophy and cerebellar ataxia, displaying altered facial structures and intellectual disability, suggesting that the truncated SNX14 mutations promote lysosomal-autophagosome dysfunction [130]. Bryant et al. recently linked SNX14 mutation to lipid metabolism dysregulation in an autosomal recessive spinocerebellar ataxia [131]. Highlighting a role for SNX14 in lipid metabolism (as discussed in section 3.2.1). No clear association between SNX13 and neurological disease, or lipid metabolism regulation has been established; still, studies with the SNX13 yeast homolog, Mdm1, point to a role in vacuole tethering which is perturbed upon the mutation of residues analogous to those identified in SNX14 that cause neuronal dysfunction, being somewhat functionally conserved [239]. SNX19, the RGS deprived PXA-RGS member, is regarded as a genetic risk factor for schizophrenia [18–20, 50], where multiple SNX19 transcripts can be of etiological relevance for this complex disorder (Fig. 4d). Curiously, SNX19 up-regulation seems to associate both with increased educational achievement and with the risk of schizophreniaThe molecular underpins are still undeciphered. SNX25, another PXA-RGS SNX, is highly expressed in the temporal lobe of patients with epilepsy [17]. Authors hypothesize that its role on epileptogenesis results from SNX25 modulation of TGF-β signaling, by shaping TGF-β receptor degradation [17]. Still, much remains unknown about SNX25-dependent protein cargoes and its brain-expression and regulation, highlighting that SNX25 role in epilepsy may result from additional molecular pathway dysregulation. Furthermore, SNX25 recent ascribed role in TrkB degradation and BDNF-TrkB signaling [68] suggests additional associations with epilepsy and also with schizophrenia, attending on BDNF and TrkB-signaling molecules on the pathophysiology of these conditions.

### SNXs Putative Role in Other Neurological Disorders — Huntington’s Disease and Amyotrophic Lateral Sclerosis

HD is autosomal dominant inherited progressive brain condition that arises from the trinucleotide repeat expansion in the huntingtin gene (Htt), causing severe cognitive, emotional and motor impairments. The relevance of a SNX-PXB, SNX21, for the Huntingtin protein intracellular trafficking regulation was recently shown [73], by orchestrating its trafficking within the endosomal population. Authors highlight SNX21/SNX20 role as scaffolds, pivotal for endosomal biology, and elaborate on SNX21 Htt and Septins interactions (Fig. 4d). Htt is a modulator of the endolysosomal network, through its association with microtubules motors, facilitating anterograde and retrograde movement of organelles. SNX21 binding to Htt is not affected (enhanced or perturbed) by the presence of polyQ pathological expansions, indicating that this interaction occurs on a normal basis and not deriving from a gain/loss of function of Htt. Still it remains unknown whether SNX21 contributes to the etiology of HD or if patients with HD display altered SNX21 levels.

ALS is a fatal progressive disease where degeneration of brain and spinal cord (SC) motor neurons (MNs) occurs, causing muscle weakness, motor impairments, speech defects, dysphasia, and ultimately death by respiratory failure. ALS pathobiology remains to be deciphered; however, a hallmark is the presence of protein aggregates within neurons. Despite being majorly sporadic, a mutation in the copper/zinc superoxide dismutase (*SOD1*) gene has been annotated in several familial cases. By studying the ALS mouse model (SOD1) of MNs neurodegeneration and iPSCs-derived MNs from ALS patients, Muzio and co-workers made evident that the retromer complex subunit — Vps35 — is downmodulated [240]. This is not surprising due to the role of retromer complex in hydrolases sorting and its significance for lysosomal health, which is notoriously disturbed in ALS. By designing an array of retromer-stabilizing drugs, authors were able to attenuate locomotion impairments and MNs survival in the ALS mice model [240]. Knowing that although Vps35 is regarded as the CRC, cargoes-recruitment and interaction also rely on proper adaptors, such as the PX-only SNX3, or the SNX-FERM SNX27, studies should be performed to assess these SNXs contributions to the ALS pathology (Fig. 4d).

Altogether, SNXs contributions to the onset or potentiation of pathological events that impact on nervous system function is increasingly growing, as a result of the multidisciplinary approaches used to functionally characterize this family. Clearly, a strong association has been made with neurodegenerative processes, particularly of those occurring in AD. In parallel links are arising to distinct neurological conditions resultant from abnormal excitatory/inhibitory control and impaired synaptic transmission, such as addiction, epilepsy, schizophrenia, ataxia, and possibly HT and ALS. This underscores the need to further assess SNXs function and particularly of SNXs-modulating drugs. Interestingly, retromer stabilizing drugs (pharmacological chaperones) have been successfully employed to stabilize the retromer CRC and to reduce Aβ in mouse hippocampal [241] and in human neurons [242].

## Concluding Remarks

Undoubtedly, unicellular, multicellular, and more complex organisms have developed a unique net of molecular players to govern the sorting of the protein content and, in this manner, tightly regulate plasma membrane protein component and how the cell/organ perceives and responds to environmental cues. SNXs are evolutionary conserved, playing sorting and signaling roles across phyla. Interestingly, only PX-BAR containing SNXs, particularly those that form the retromer complex, are present in less evolved organisms. Throughout speciation, SNXs containing other domains emerged, with roles in protein-protein, protein-lipid interactions and in signaling. More so, upon the appearance of nerve cells, and of a nervous system, the complexity within this family markedly increased, which mirrors its significance for nervous system homeostasis regulation. Thus, the observed associations between SNXs dysregulation and conditions that affect the nervous system function and, ultimately, health and lifespan are not surprising. Whether SNXs (dys)regulation are directly or indirectly shaping nervous system homeostasis is still debatable, although causative links have been established.

In this review, we have detailed a historical evolutionary retrospective of the SNXs development as a family, and that of its members association with nervous system function, namely in (neuro)developmental, metabolic, and plasticity processes. In addition, we made an effort to compile a comprehensive review of the known roles of SNXs within the nervous system, particularly in the etiology and/or progression of several neurological conditions. We covered aspects of endocytic trafficking (dys)regulation, caused by SNX mutation and/or expression up- or down-modulation in disease-linked proteins, in an effort to scrutinize the molecular mechanisms underpinning SNX association with neurological conditions. We envision that this association will continue to grow, as distinct biochemical, bioinformatic, and structural approaches, among others, are designed and improved, enabling a more accurate identification of SNXs binding partners, and their function within the nervous system. Although trafficking regulation in neurological disorders is undoubtedly a complex process, in which several effector and adaptor proteins cooperate, the characterization of common sorting defects in the neuropathology of these conditions caused by SNXs can anticipate unique molecular pathways prone to be therapeutically modulated. In fact, upon the clarification of the multiplicity of SNXs actions within nervous system regulation, new pharmacological approaches can be undertaken to ameliorate several neurological conditions such as neuropsychiatric and neurodegenerative disorders.

## Supplementary Information

ESM 1 (DOCX 27 kb)

## Data Availability

Not applicable.

## Abbreviations

AMPA: Amino-3-hydroxy-5-methyl-4-isoxazolepropionic acid
Aβ: Amyloid beta
AD: Alzheimer’s disease
APP: Amyloid precursor protein
BAR: Bin-amphiphysin-rvs
BBB: Blood–brain barrier
CC: Coiled-coil domain
CCC: COMMD/CCDC22/CCDC93 complex
CI-MPR: Cation-independent mannose-6-phosphate receptor
CNS: Central nervous system
CPY: Carboxy peptidase Y
CRC: Cargo recognition complex
CSF: Cerebrospinal fluid
DAG: Diacylglycerol
DGKζ: Diacylglycerol kinase ζ
DMT1: Divalent metal transporter 1
DS: Down’s syndrome
EGFR: Epidermal growth factor receptor
FERM: Protein 4.1/ezrin/radixin/moesin
FHA: Forkhead association domain
GABA: *γ*-Aminobutyric acid
GPCRs: G protein–coupled receptors
GLUT1: Glucose transporter 1
GluR1: Glutamate receptor 1
GluR2: Glutamate receptor 2
IDE: Insulin-degrading enzyme
ILV: Intraluminal vesicle
KIN: Kinesin associated domain
LRP1: Low-density lipoprotein receptor
LTP: Long-term potentiation
mGluRs: Metabotropic glutamate receptors
MIT: Microtubule associated domain
MCT: Monocarboxylate transporter
MNs: Motor neurons
NMDAR: N-methyl-D- aspartate receptors
NLG: Neuroligin
PD: Parkinson’s disease
PDZ: PSD-95, Disc-large and ZO-1
PIP: Phosphoinositide
PSD: Postsynaptic density
PX: PX domain
PXA: PX associated domain A
PXB: PX associated domain B
PXC: PX associated domain C
RGS: Regulator of G protein signaling domain
SC: Spinal cord
SH3: Src Homology 3
SMA-6: Type I TGF-β receptor
SNX: Sorting nexin
SORLA: Sortilin
StxB: Shiga-like toxin β subunit
TCA: Tricarboxylic acid cycle
TGN: Trans-Golgi network
VPS: Vacuolar protein Sorting
Wash1: Wiskott-Aldrich syndrome homologue 1
Wls: Wntless
