# Supplementary material for: Sorting Out Sorting Nexins Functions in the Nervous System in Health and Disease
Source: Mol Neurobiol. 2021 May 1;58(8):4070–106. doi: 10.1007/s12035-021-02388-9 (PMC8280035; doi:10.1007/s12035-021-02388-9)
Supplement: Supplementary file 1 — (DOCX 27 kb) [file 12035_2021_2388_MOESM1_ESM.docx]

Supplementary Information - Progress in neurobiology REVIEW - 2019.9.9

**Table 1.** List of the representative model organisms used to search for phylogenetic relationships between predicted SNXs.

| **Organism** | **Accession number/bioproject of representative genome/assembly** | **Number of proteins downloaded** |
| --- | --- | --- |
| *Escherichia coli* | NC_000913.3 | 4242 |
| *Chlamydomonas reinhardtii* | PRJNA21061 | 14488 |
| *Saccharomyces cerevisiae* | PRJNA128, PRJNA43747 | 6002 |
| *Arabidopsis thaliana* | GCA_000001735.2, RJNA116, PRJNA10719, PRJNA11796, PRJNA13191 | 48265 |
| *Amphimedon queenslandica* | GCA_000090795.1,PRJNA66531, PRJNA39517 | 24115 |
| *Trichoplax adhaerens* | GCA_000150275.1, PRJNA30931, PRJNA12874 | 11520 |
| *Hydra vulgaris* | ACZU00000000.1 | 21990 |
| *Mnemiopsis leidyi* | Available outside NCBI (https://research.nhgri.nih.gov/mnemiopsis/) | 16548 |
| *Aplysia californica* | GCA_000002075.2, PRJNA209509, PRJNA13635 | 27591 |
| *Caenorhabditis elegans* | assembly WBcel235 | 28420 |
| *Drosophila melanogaster* | assembly Release 6 plus ISO1 MT | 30493 |
| *Danio rerio* | assembly GRCz11 | 52829 |
| *Xenopus laevis* | assembly Xenopus_laevis_v2 | 56667 |
| *Mus musculus* | assembly GRCm38.p6 | 76226 |
| *Rattus norvegicus* | assembly Rnor_6.0 | 56110 |
| *Homo sapiens* | assembly GRCh38.p12 | 113620 |

**Methodology:**

Two distinct methodologies were used, in order to guarantee the presence of the conserved domains associated with SNX family and to exclude other characterized PX-domain-containing proteins.

Using Biopython and makeblastdb (an application from NCBI to create BLAST databases, version 2.9.0+) we built a local database containing the proteome of the 16 organisms (Table 1). We performed BLAST searches on the database using 34 queries. Those queries corresponded to the PX domain region of the 34 SNX identified in humans. By using the PX region only we did not limit the search to known SNXs but expanded it to search for other proteins containing the PX domain. Simultaneously, it allowed to search for uncharacterized PX-domain-containing proteins that could be SNXs in other organisms. These results were further filtered to exclude non-SNX PX-domain-containing proteins. We restricted the results to those displaying an e-value below 10^-3^ in the BLAST search. As a checkpoint for the unbiasing nature of the analysis most of the different human SNXs appeared several times as a result in the various BLAST searches, including other human PX-domain-containing proteins. We obtained nearly 400 different results across the 16 organisms that were analysed further. The various results were then analysed using two methodologies. First, we checked the obtained results for conserved domains using the CDD website from NCBI (https://www.ncbi.nlm.nih.gov/cdd/) and Prosite (https://prosite.expasy.org/). All sequences contained the PX domain as expected. If other domains were present, we analysed if they were associated with sorting nexins or other characterized PX-domain-containing proteins. As a second approach, the different results were submitted to a BLAST against the full NCBI protein database in order to check if the most similar results were either SNXs or other PX-domain-containing proteins. Furthermore, all alternative isoforms within a proteome were excluded (proteins obtained from the same physical genomic location) and in the case of *Xenopus laevis*, that display polyploidy, one of the two homologs were excluded. A total of 293 sequences were used in the final analysis (table 2). As the only common segment between the 293 sequences was the PX domain, this segment was extracted from each sequence to be phylogenetically analysed.

**Table 2.** List of the sequences used for the phylogenetic analyses.

| Organism | Sequences used in the analysis |
| --- | --- |
| *Homo sapiens* | NP_003090, XP_016864486, NP_003091, XP_016870205, NP_689973, NP_695003, NP_003785, NP_689413, NP_689419, XP_016856917, NP_037453, NP_057308, NP_443180, XP_016878417, NP_899229, NP_878274, NP_219489, NP_115543, NP_055563, NP_001317652, NP_689841, NP_037438, NP_078980, NP_001337791, NP_722523, XP_024304289, NP_001304710, NP_037454, NP_689450, NP_037478, NP_071416, NP_079074, NP_054754, NP_003786 |
| *Mus musculus* | NP_062701, NP_570614, NP_080662, NP_766056, NP_001019731, NP_780692, NP_542124, NP_001186117, NP_081274, NP_001177085, NP_758481, NP_079940, XP_006539951, XP_011244931, NP_080107, NP_082116, NP_598685, XP_006522729, NP_710147, NP_001075953, NP_079988, NP_081188, NP_001074602, XP_006515125, XP_006511246, NP_083150, NP_997096, NP_082311, NP_083241, NP_001103780, NP_083344, NP_001020783, NP_083670, NP_059500 |
| *Rattus novergiccus* | NP_445863, NP_001101122, NP_001099605, NP_001100121, XP_006231002, XP_006243185, NP_001121022, XP_006235181, NP_001102181, NP_001012083, NP_001099382, NP_001121109, XP_008757475, XP_008765788, NP_001020890, NP_001020170, XP_008760780, NP_001102996, NP_001011981, NP_001103621, XP_003750386, NP_001019923, XP_006235174, XP_006240084, NP_001101644, NP_001101601, XP_224863, NP_001013103, NP_001186098, NP_001102287, NP_071625, NP_001100302, NP_001008365, NP_001037748 |
| *Xenopus laevis* | XP_018110059, NP_001086819, XP_018119692, NP_001089520, XP_018108412, XP_018091100, NP_001088935, XP_018087703, NP_001084945, XP_018091848, XP_018120748, XP_018083848, XP_018094802, XP_018123237, XP_018113289, XP_018093872, XP_018091805, NP_001080742, XP_018089103, NP_001086730, NP_001090475, XP_018118291, XP_018122869, NP_001085023, XP_018083166, XP_018085890, NP_001086605, NP_001087475, NP_001079514, XP_018119773, XP_018123587, XP_018109657, XP_018119689 |
| *Danio rerio* | NP_001122143, NP_001028907, NP_001093476, NP_001015063, NP_001032787, NP_001017798, NP_001116327, NP_001014368, NP_999934, NP_001025388, NP_001002229, NP_001313419, XP_009304805, NP_001025295, XP_005158779, XP_005158459, NP_001071052, XP_694713, NP_001070836, XP_005163245, XP_693885, NP_001038622, NP_956834, NP_001038565, NP_001104707, XP_009292601, XP_021323912, NP_001038258, XP_697433, NP_001124114, NP_001156765, XP_009289456, NP_001132934, NP_001103866, NP_001037781, NP_957417, NP_991228, NP_001038839, NP_001028903, NP_001032183 |
| *Drosophila melanogaster* | NP_648348, NP_608777, NP_609199, NP_610002, NP_608709, NP_609607, NP_609353, NP_727023, NP_524532, NP_572421, NP_650214, NP_611252 |
| *Caenorhabbditis elegans* | NP_001294088, NP_508216, NP_001256763, NP_001024683, NP_001076762, NP_492758, NP_503026, NP_496844, NP_492437 |
| *Aplysia californica* | XP_005103389, XP_005105399, XP_012943166, XP_012944347, XP_005098618, XP_012939843, XP_012935881, XP_012940928, XP_005096793, XP_005108639, XP_005096824, XP_005098481, XP_012936774, XP_005089468, XP_012936261, XP_012936489, XP_005098033, XP_005088897, XP_012934709, XP_005112274 |
| *Mnemiopsis leidyi* | ML02636a, ML04816a, ML000117a, ML20256a, ML015632a, ML138312a, ML083024a, ML014426a, ML06163a, ML015726a, ML073280a, ML274414a, ML065748a, ML30619a |
| *Hydra vulgaris* | XP_002156985, XP_004206349, XP_012560278, XP_012558573, XP_002157161, XP_004206209, XP_012563699, XP_002165900, XP_012566882, XP_012564083, XP_012566573, XP_002155868, XP_012558014, XP_012560002, XP_012557028, XP_012560700, XP_012558254, XP_012556217, XP_012553664, XP_012557629, XP_002162612, XP_012557246 |
| *Trichoplax adhaerens* | XP_002108019, XP_002111028, XP_002109479, XP_002113191, XP_002115328, XP_002116630, XP_002110074, XP_002111499, XP_002109193, XP_002110221, XP_002109195, XP_002112504, XP_002116983, XP_002110844 |
| *Amphimedon queenslandica* | XP_003382576, XP_003384967, XP_003386746, XP_019853969, XP_019849679, XP_019858797, XP_019849649, XP_019853027, XP_019849243, XP_003386341, XP_003388361, XP_011403125, XP_019852719, XP_003387890, XP_003386271 |
| *Arabidopsis thaliana* | NP_196232, NP_200652, NP_179190, NP_001185002 |
| *Saccharomyces cerevisiae* | NP_014712, NP_012498, NP_013717, NP_010170, NP_015002 |
| *Chlamydomonas reinhardtii* | XP_001695319, XP_001701189, XP_001690659 |
| *Escherichia coli* | - |

The dataset of 293 PX domain sequences were aligned using CLUSTALW within MEGA7 [1]. MEGA7 was used to reconstruct the phylogeny of SNXs using maximum likelihood with Gamma distributed rates (5 categories). 500 replications were performed for a bootstrap analysis. Furthermore, FigTree was used to display the obtained phylogeny and to colour the different clades. As *Danio rerio*, *Xenopus laevis*, *Mus musculus*, *Rattus norvegicus* and *Homo sapiens* display a similar composition of SNXs we used the clades commonly established by the homologues in five organisms to indicate the different SNXs in the figure in a secure way. Some of the SNXs formed sister clades in these five organisms (for example SNX10 and SNX11) with the other organisms displaying homologues phylogenetically related to both.

1. Kumar, S., G. Stecher, and K. Tamura, *MEGA7: Molecular Evolutionary Genetics Analysis Version 7.0 for Bigger Datasets.* Mol Biol Evol, 2016. **33**(7): p. 1870-4.
